# Laser Ultrasonic Detection and Signal Enhancement of Internal Microdefects in LPBF Ti6Al4V with Anisotropic Microstructure: Simulations and Experiments

**DOI:** 10.3390/mi17091025

**Published:** 2026-08-28

**Authors:** Xingyu Zhou, Jia Xie, Yixuan He, Ping Hu

**Affiliations:** 1 School of Power and Mechanical Engineering, Wuhan University, Wuhan 430072, China; iniesta125@163.com; 2 Xi’an Aerospace Mechatronics and Intelligent Manufacturing Co., Ltd., Xi’an 710100, China; jiajiaxie666@163.com

**Keywords:** laser ultrasonics, Laser Powder Bed Fusion (LPBF), anisotropic microstructure, defect detection, signal processing (PCA-SAFT)

## Abstract

Laser Powder Bed Fusion (LPBF) has revolutionized high-end manufacturing, particularly in aerospace and biomedical fields. However, internal defects such as pores, cracks, and inclusions compromise the structural integrity and service reliability of LPBF components. Laser ultrasonics, a non-contact, broadband non-destructive testing (NDT) method, offers a promising solution for detecting and characterizing these defects. This study systematically investigated laser ultrasonic testing technology for LPBF-fabricated Ti6Al4V using a combined approach of physics-driven simulation modeling and experimental validation. To accurately model material anisotropy, a finite element model was developed that integrated Voronoi algorithm-generated polycrystalline microstructures with orientation-dependent elastic tensors, providing a comprehensive representation of the material’s microstructural heterogeneity. Simulation results revealed that while sub-100-μm defects yield weak ultrasonic scattering signals, the Synthetic Aperture Focusing Technique (SAFT) markedly improves the detection and imaging performance for such small-scale defects. Experimental validation using a laser ultrasonic system identified a 90 μm internal defect in the LPBF Ti6Al4V specimen, though a 75 μm defect was undetectable. This highlights the need for enhanced sensitivity. A signal processing method combining time-truncation principal component analysis (PCA) with targeted noise reduction and SAFT was proposed to reduce high-frequency noise and improve high-resolution imaging, enhancing defect detection accuracy. This study provides theoretical foundations and technical support for high-precision defect detection in metal additive manufacturing components, with significant implications for quality control in high-end equipment manufacturing.

## 1. Introduction

Laser Powder Bed Fusion (LPBF), a core additive manufacturing (AM) technology, has revolutionized manufacturing by overcoming traditional constraints on complex geometries through its ‘discrete-deposition’ layer-wise fabrication principle. Enabled by digital design and near-net-shaping capabilities, LPBF is now widely applied in aerospace, biomedical engineering, and high-value manufacturing sectors [1,2]. However, the LPBF process involves intricate solid–liquid–gas phase transformations and coupled multi-physics field interactions, where heterogeneous heat input, powder impurities, and environmental fluctuations collectively induce microdefects including pores, micro-cracks, and incomplete fusion. Defined by their size distribution, spatial morphology, and evolutionary behavior, these microstructural anomalies directly dictate material fatigue failure mechanisms and significantly impair component service life and mechanical performance [3]. For instance, intergranular cracks in LPBF-fabricated Haynes 230 alloy drastically reduce tensile strength and ductility [4], while increasing porosity systematically impairs load-bearing capacity and structural reliability by compromising stress distribution homogeneity [5,6]. This highlights the critical need for developing high-precision non-destructive testing (NDT) techniques to enable real-time in situ monitoring and full-life-cycle quality control of AM components.

**Figure 1:**
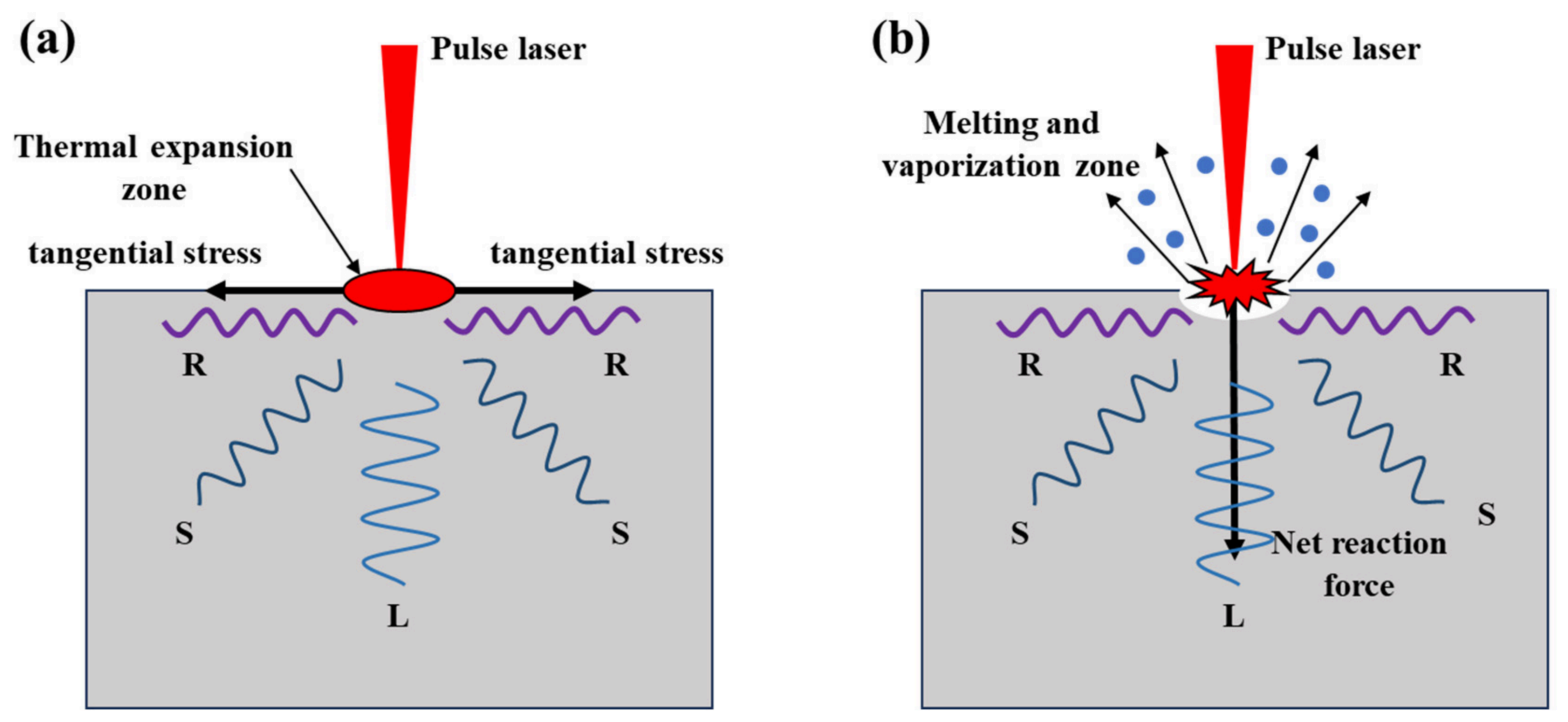
The schematic diagram of principles of laser-induced ultrasonic waves: (**a**) thermoelastic mechanism and (**b**) thermal ablation mechanism.

**Figure 2:**
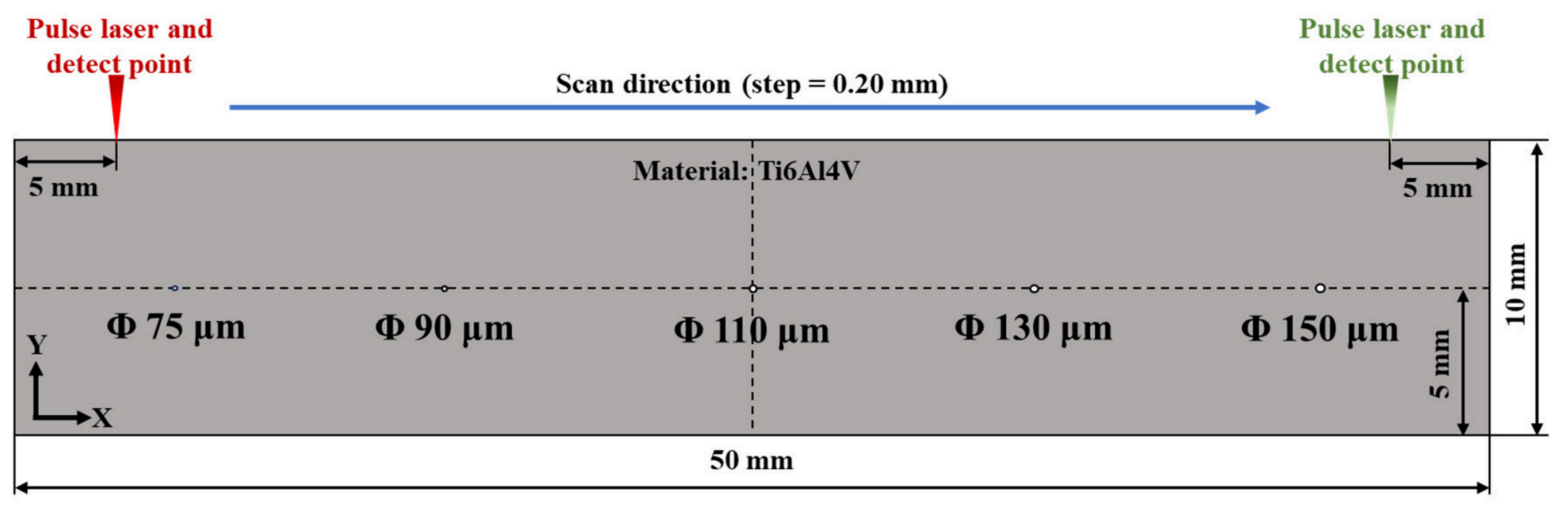
Laser ultrasonic simulation model.

Among diverse inspection technologies, laser ultrasonics stands out for its unique advantages. As a non-contact, wide-bandwidth, high-precision non-destructive testing (NDT) technique, it replaces conventional ultrasonic piezoelectric sensors with a laser optical system [7]. Laser ultrasonics primarily relies on two excitation mechanisms: (1) the non-destructive thermoelastic mechanism, in which low-energy pulsed laser irradiation induces localized thermal expansion to generate ultrasonic waves (Figure 1a); (2) the thermo-ablation mechanism, where laser energy exceeding the material damage threshold excites ultrasonic waves, though this causes minor surface damage (Figure 1b). Under both mechanisms, lasers could simultaneously generate longitudinal (L), shear (S), and Rayleigh (R) waves within the materials. Laser ultrasonic testing involves irradiating samples with continuous/pulsed laser light; scattered/reflected light carries ultrasonic information, which is collected and analyzed by an optical detection system. In 1963, White [8] first demonstrated pulsed laser excitation of elastic waves on material surfaces, and in 1968, he and Lee [9] used lasers to excite Rayleigh waves. This pioneering work laid a solid physical foundation for laser ultrasonics (LU) technology. With the in-depth analysis of laser–matter interaction mechanisms, innovations in optical interferometric detection, and high-performance signal processing algorithms, LU has evolved from a theoretical concept into a mature NDT method [10].

Numerous studies have explored laser ultrasonics for detecting internal defects in additively manufactured parts. Early work primarily leveraged its wide bandwidth and multimodal characteristics, using conventional A/B/C-scans to validate defect detection capability in additively manufactured alloys. Davis et al. performed laser ultrasonic testing on selective laser melting (SLM) parts, accurately identifying 2 mm-diameter internal voids [11]. Yu et al. assessed internal voids in additive-manufactured Ti6Al4V parts, obtaining clear B-scan images of sub-millimeter-scale defects (minimum 0.8 mm in diameter) [12]. Liu et al. successfully detected internal 90 μm-diameter internal voids in additively manufactured Ti6Al4V, with clear A/C-scan images for identification. These studies demonstrated the potential of LU methods for detecting internal microdefects [13]. However, detecting sub-millimeter internal defects in additively manufactured metals is far more challenging than surface/near-surface defects. First, ultrasonic scattering from internal microdefects is primarily the Rayleigh type, with echo signal energy being a minuscule fraction of incident wave energy. To address this, current research has introduced imaging algorithms to enhance defect signals, enabling high-resolution visualization. Mei et al. combined full-matrix capture with deep learning for complex internal defects, demonstrating excellent imaging of defect shape, size, and location [14]. Marchese et al. improved preprocessing and phase migration algorithms for laser ultrasonic B-scans, reconstructing defect images in Ti6Al4V and AlSi10Mg parts, to detect 200 μm blind bottom holes [15]. Liu et al. used multi-mode laser ultrasonic frequency-domain synthetic aperture focusing technology (F-SAFT) and the frequency-domain total focusing method (F-TFM) to image 0.3 mm internal transverse holes in aluminum alloys [16]. Dai et al. characterized internal defects in bimetallic composites by optimizing the synthetic aperture focusing algorithm and combining frequency-domain synthetic aperture focusing technology with multimodal laser ultrasonic information [17]. Liu et al. proposed a laser ultrasonic multimodal sparse array imaging method based on dynamic multi-particle swarm optimization, enabling non-destructive evaluation of 0.3 mm in diameter and 5.5 mm in depth side-drilled holes in aluminum alloys with excellent imaging performance [18].

Furthermore, additive manufactured alloys often form coarse grains during solidification, whose elastic anisotropy induces intense grain scattering noise. This microstructure noise and weak scattering signals from internal microdefects overlap significantly in both the time and frequency domains, severely reducing the signal-to-noise ratio (SNR) and defect signal distinguishability. Early scattering attenuation models were primarily designed for simple polycrystalline media with cubic crystal systems, equiaxed grains, and single-phase structures, falling to account for such complex structures [19]. With growing focus on realistic complex polycrystalline structures, such as elongated, duplex, or multiple-phase grains formed by casting, welding, and additive manufacturing, researchers have developed corresponding ultrasonic backscattering models for characterizing material microstructures [20,21,22]. Peng et al. used ultrasonic NDT to evaluate the anisotropy of additively manufactured 316L, demonstrating that the microstructure causes significant scattering attenuation [23]. To mitigate grain noise, denoising algorithms are typically employed to improve ultrasonic imaging resolution. Wang et al. proposed a variational mode decomposition (VMD)-based denoising method, using the genetic algorithm and weighted permutation entropy to optimize the VMD parameters for signal reconstruction [24]. Applied to the laser ultrasonic detection of 1 mm internal defects in aluminum alloys, this method outperformed conventional denoising algorithms. He et al. proposed a post-processing algorithm for laser ultrasonic SAFT imaging, improving image SNR and enabling detection of 0.5 mm-diameter, 15 mm-deep sub-millimeter defects [25]. Additionally, additively manufactured components inherently exhibit uneven surface roughness, which enhances mode conversion between bulk and surface waves, introducing additional bulk wave noise that interferes with internal defect assessment. Recent studies have analyzed surface roughness impacts on laser ultrasonic NDT. Zeng et al. showed via A/B-scan images that surface roughness significantly reduces SNR in the internal through-hole detection [26]. Yin et al. combined finite element simulations and experiments to investigate the surface roughness effects, successfully detecting 0.6 mm-diameter internal through-hole in additively manufactured 316L [27]. Liu et al. proposed regularized expectation-maximization clustering based on the laser ultrasonic imaging method, enabling detection of 0.4 mm defects even under high surface roughness conditions [28]. Finally, for slender components with defects near material boundaries, ultrasonic signal blind zones arise from overlapping generation waves and boundary echoes, hindering microdefect signal identification. Huang et al. proposed a signal processing method combining principal component subtraction (PCS) with synthetic aperture focusing technique (SAFT), suppressing boundary echo and surface wave interference in picosecond laser ultrasonic defect detection [29]. Complementing this, recent photoacoustic imaging research has demonstrated that two-dimensional surface acoustic field measurements paired with adaptive inversion algorithms enable accurate reconstruction of three-dimensional wave propagation and backpropagation [30]. While laser ultrasonics shows great promise for additive manufacturing defect detection, microdefect detection in LPBF-manufactured parts faces significant challenges, including unclear detection mechanisms and lack of theoretical guidance for wave field interpretation and defect visualization.

This paper presents a systematic investigation into laser ultrasonic non-destructive testing (NDT) and imaging algorithms for the detecting internal microdefects in laser powder bed fusion (LPBF) alloys. First, finite element (FE) simulations are employed to validate the feasibility of laser ultrasonic testing for detecting internal microdefects, with a synthetic aperture focusing algorithm applied to optimize simulation result imaging. Second, a polycrystalline simulation model incorporating grain orientation anisotropy is developed to replicate the complex microstructural characteristics of additively manufactured metals, enabling analysis of grain scattering effects on ultrasonic propagation. Finally, a hybrid method integrating principal component analysis (PCA) and synthetic aperture focusing techniques (SAFT) is proposed for laser ultrasonic testing of LPBF-manufactured Ti6Al4V alloys with sub-millimeter-scale internal microdefects. This study provides a theoretical foundation and technical support for the high-precision detection of internal microdefects in metal additive manufacturing components.

## 2. Laser Ultrasonic Finite Element Simulation

### 2.1. Simulation Model Development

To thoroughly investigate the interaction mechanism between laser-induced ultrasonic waves and submillimeter-scale defects, and to verify the sensitivity of this method for detecting microdefects, a finite element model of laser-induced ultrasonic waves was first established using a simulation platform, as illustrated in Figure 2. The model geometry is a 50 mm × 10 mm rectangular domain. Five hole defects of varying diameters (75 μm, 90 μm, 110 μm, 130 μm, and 150 μm) were pre-defined at equal intervals (9 mm between adjacent centers) along the horizontal direction, located 5 mm below the upper surface to simulate micro-damage within the material.

To simplify the model, a Hanning-window-modulated sinusoidal pulse is utilized to simulate the generation of the initial laser ultrasound. This is mathematically expressed as follows:

(1) $$f(t)=\left\{\begin{array}{cc} \left[1-\cos(2\pi f_{0}t)\right]\sin(2\pi f_{0}t), & 0\leq t\leq 1/f_{0}\\ 0\;\;\;\;\;\;\;\;\;\;\;\;\;\;\;\;\;\;\;, & t>1/f_{0} \end{array}\right.$$

where $f_{0}$ represents the ultrasonic frequency, which is set to 5 MHz. To simulate the generation of laser-induced ultrasonic waves, the Solid Mechanics module in the simulation software is employed to apply a boundary load with a radius of 0.5 mm to the upper boundary of the model. The governing equations for the propagation of ultrasonic waves in solid materials are as follows:

(2) $$\rho \frac{\partial^{2}u}{\partial t^{2}}=\left(\lambda +\mu \right)\nabla \left(\nabla \cdot u\right)+\mu \nabla^{2}u+F$$

where ρ is the material density, u is the displacement vector during deformation, λ and μ are Lamé constants, and F is the mechanical load vector. To simulate the reflection of ultrasonic waves at the backwall of the object, a fixed boundary condition is applied at the ultrasonic reflection interface, specifically, the lower boundary of the model:

(3) u=0

To simulate the laser scanning inspection process, a laser scan simulation was conducted across a 40 mm section of the model’s upper surface from left to right at equal intervals with a step size of 0.20 mm. At each position, both the generation and the detection lasers were co-located. The simulation was set to a total duration of 4 μs, with a time step of 0.008 μs. The material used for the simulation was Ti6Al4V, with the relevant physical parameters provided in Table 1.

### 2.2. Defect Detection Simulation

**Figure 3:**
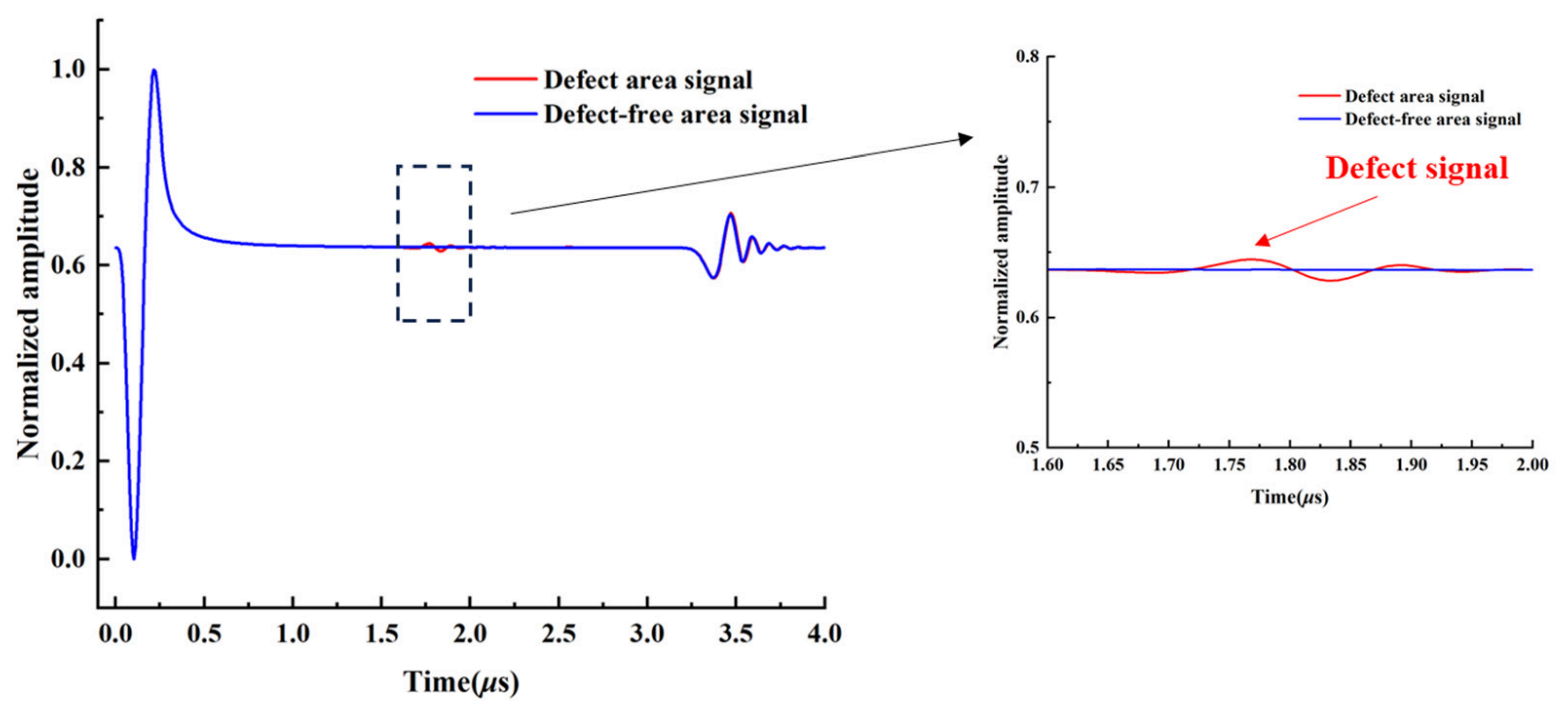
Simulated A-scan signals for defect and defect-free areas.

The ultrasonic A-scan signals generated from the defect detection simulation are presented in Figure 3, including waveforms from defective and defect-free regions. Within the 4 μs measurement window, two distinct waveform components are observed. A high-amplitude initial excitation wave appears at approximately 0.3 μs, directly mirroring the temporal envelope of the input boundary load. A lower-amplitude backwall reflection arrives at around 3.7 μs, having undergone significant amplitude attenuation and waveform distortion during propagation due to material absorption, grain boundary scattering, and multimode conversion. By comparing signals from defective and defect-free regions, a faint defect scattering signal is revealed at approximately 1.8 μs upon local magnification of the defect waveform.

**Figure 4:**
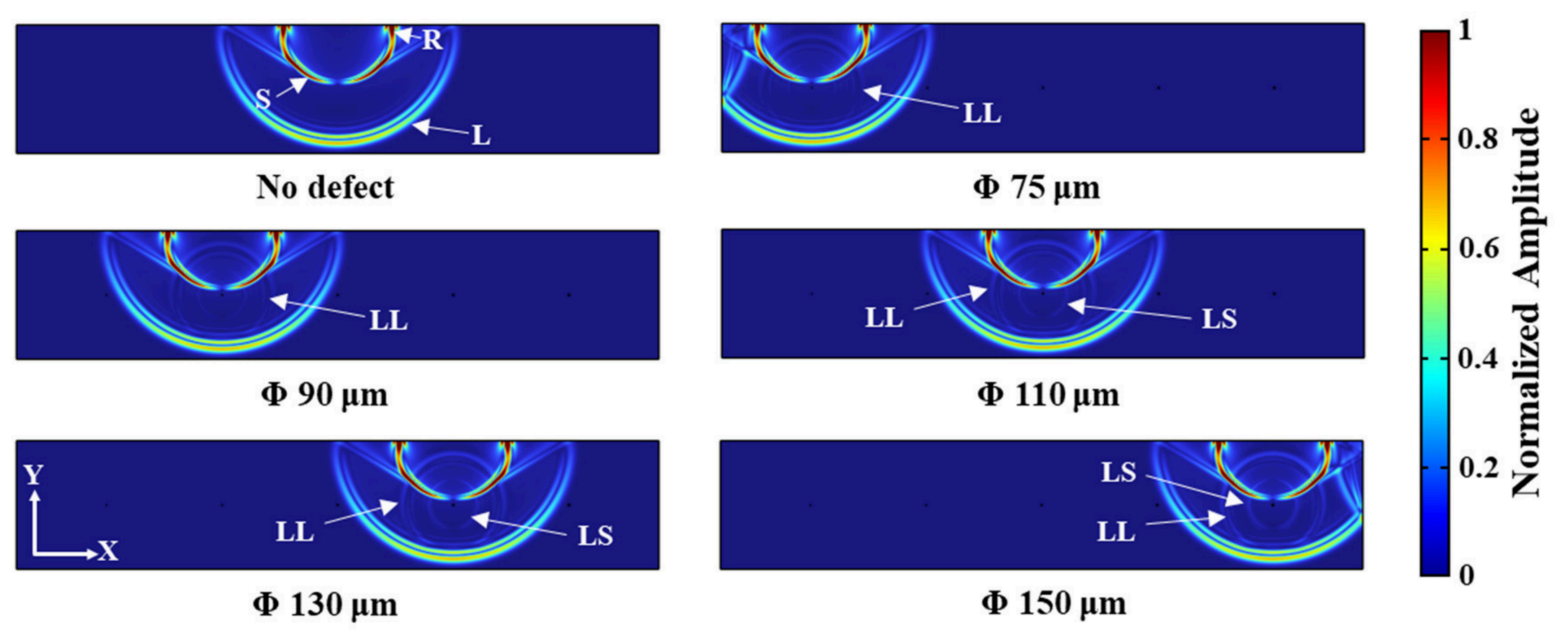
Laser-ultrasonic FEM simulation: displacement distribution at *t* = 1.6 μs.

The ultrasonic displacement distribution at *t* = 1.6 μs, as obtained from the simulation, is illustrated in Figure 4. This full-field snapshot captures instantaneous wave propagation across the entire cross-section, enabling clear differentiation of wave types based on distinct phase velocities and propagation paths. From the ultrasonic displacement image of the defect-free model in Figure 4, it is evident that the laser simultaneously excites longitudinal (L), transverse (S), and Surface/Rayleigh (R) waves within the material. The R-wave propagates along the material surface, whereas the L-wave and S-wave propagate into the material interior, making body waves suitable for detecting internal defects. As depicted in Figure 4, when the incident ultrasonic signal encounters a defect, part of its energy is scattered by the defect, while the remaining energy is transmitted through it. The figure clearly demonstrates a mode conversion phenomenon at the defect site, where the incident longitudinal wave transforms into scattered longitudinal waves (Longitudinal–Longitudinal, LL) and scattered shear waves (Longitudinal–Shear, LS). Specifically, LL waves propagate around the defect at the speed of longitudinal waves and exhibit a larger amplitude. In contrast, LS waves propagate at a slower speed than LL waves, traveling outward at the speed of shear waves. They appear darker in color compared to LL waves and have a smaller amplitude. A comparison of the acoustic field maps for defects of different sizes reveals that as the defect diameter decreases, the energy of the LL and LS waves generated by the modal transition gradually weakens. Particularly, when the defect size is 75 μm and 90 μm, the LS waves become difficult to observe in the ultrasonic displacement distribution map.

**Figure 5:**
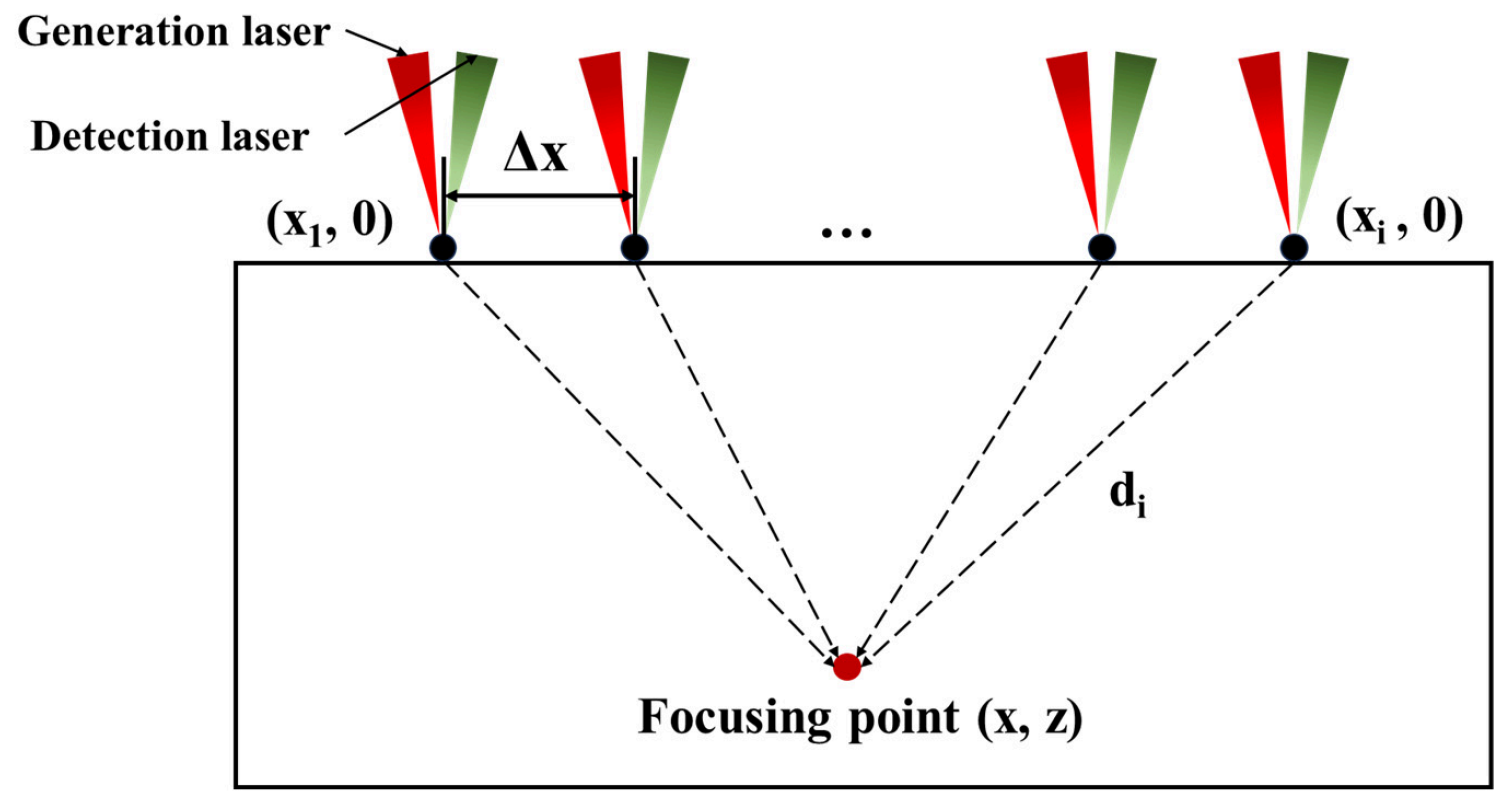
Schematic diagram of the synthetic aperture focusing technique (SAFT) principle.

The analysis of the A-scan signals and displacement images indicates that, since the defect is centrally located in the material, its reflection signal consistently appears between the generation wave and the backwall wave, with a relatively weak amplitude. Consequently, focusing on the region between the generation wave and the backwall wave (i.e., the backscatter signal region) for synthetic aperture focusing technique (SAFT) imaging can effectively enhance the contrast of images derived from the defect signal. SAFT employs time-delayed superposition of received signals from various positions to achieve focused imaging of the inspection area. This method effectively improves the image’s lateral resolution and SNR of the images [33]. The principle of SAFT is illustrated in Figure 5. The ultrasonic propagation time from the imaging point to each signal reception position is calculated as follows:

(4) $$t_{i}=\frac{2\sqrt{z^{2}+{\left(x-x_{i}\right)}^{2}}}{c}=\frac{2d_{i}}{c}$$

where $d_{i}$ is the distance from the scanning point to the focal point, and *c* is the wave speed. The intensity $I\left(x,z\right)$ at the focal point can be obtained by summing the signals $S_{i}$ from each point on the scanning surface at time *t*, and is expressed as follows:

(5) $$I(x,z)=\sum_{i=1}^{n}S_{i}\left(t_{i}\right)$$

Ultrasonic wave velocity stands as a foundational parameter for SAFT imaging, directly dictating spatial resolution, defect localization precision, and quantitative reconstruction accuracy. This critical parameter exhibits dual dependency. It is intrinsically tied to the intrinsic mechanical properties of materials. It is also dynamically modulated by extrinsic environmental factors and intrinsic microstructural characteristics. For isotropic metallic materials utilized in the SAFT simulations framework, longitudinal wave velocity is derived from fundamental elastic constants using the following equation:

(6) $$c_{L}=\sqrt{\frac{E\left(1-\nu \right)}{\rho \left(1+\nu \right)\left(1-2\nu \right)}}$$

Here, $c_{L}$ denotes the longitudinal wave velocity, with all associated material mechanical properties tabulated in Table 1. The calculated isotropic longitudinal wave velocity of 6072 m/s is employed as the core input parameter for SAFT-based ultrasonic image reconstruction of simulation datasets.

**Figure 6:**
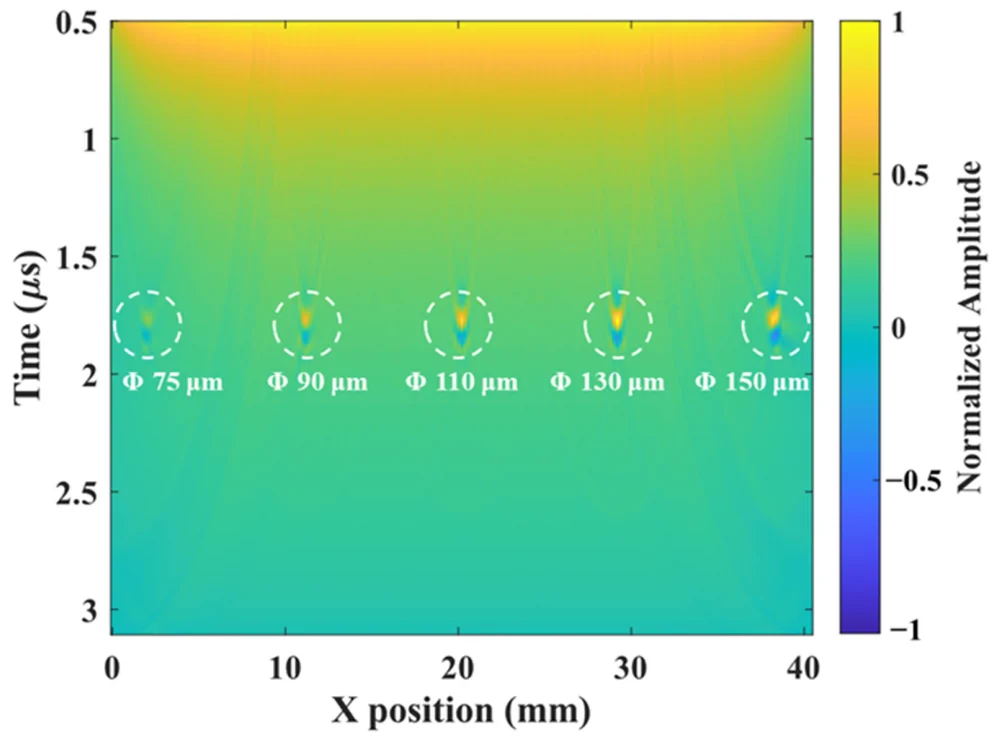
SAFT image derived from the finite element simulation results.

Laser ultrasonic scan data collected over a 40 mm length on the upper surface of the model with a step size of 0.20 mm was utilized for SAFT calculations. The resulting SAFT image, depicted in Figure 6, clearly reveals five sets of defect signals. Notably, the intensity of these signals decreases as the defect size diminishes, thereby validating the effectiveness of laser ultrasonics in detecting microdefects within additively manufactured metals. This demonstrates the potential of this technique for non-destructive evaluation in advanced manufacturing processes, emphasizing its capability to provide detailed insights into material integrity.

### 2.3. Microstructural Finite Element Simulation

In practical laser ultrasonic testing, ultrasonic waves scatter at grain boundaries due to the anisotropy of grain orientations in additively manufactured metals. The scattering can result in severe grain noise, which may mask signals from microdefects. Consequently, it is crucial to use numerical simulation methods to assess the impact of grain noise on laser ultrasonic testing signals. To model grain structures while managing computational resources efficiently, a two-dimensional Voronoi algorithm was employed. This algorithm divides a plane into convex polygonal cells by randomly generating points on the plane. Each cell is defined such that the distance from any point within it to its generating point is less than the distance to any other generating point. By adjusting the distribution of generating points and random parameters, the simulation can produce grain geometric models with varying sizes, shapes, and distribution patterns. In the two-dimensional space X, any generated point within a cell $R_{k}$ satisfies the following condition [34]:

(7) $$R_{k}=\underset{i\neq k}{\cap }\left\{\left.x\in X\right|d\left(x,P_{k}\right)\leq d\left(x,P_{i}\right)\right\}$$

**Figure 7:**
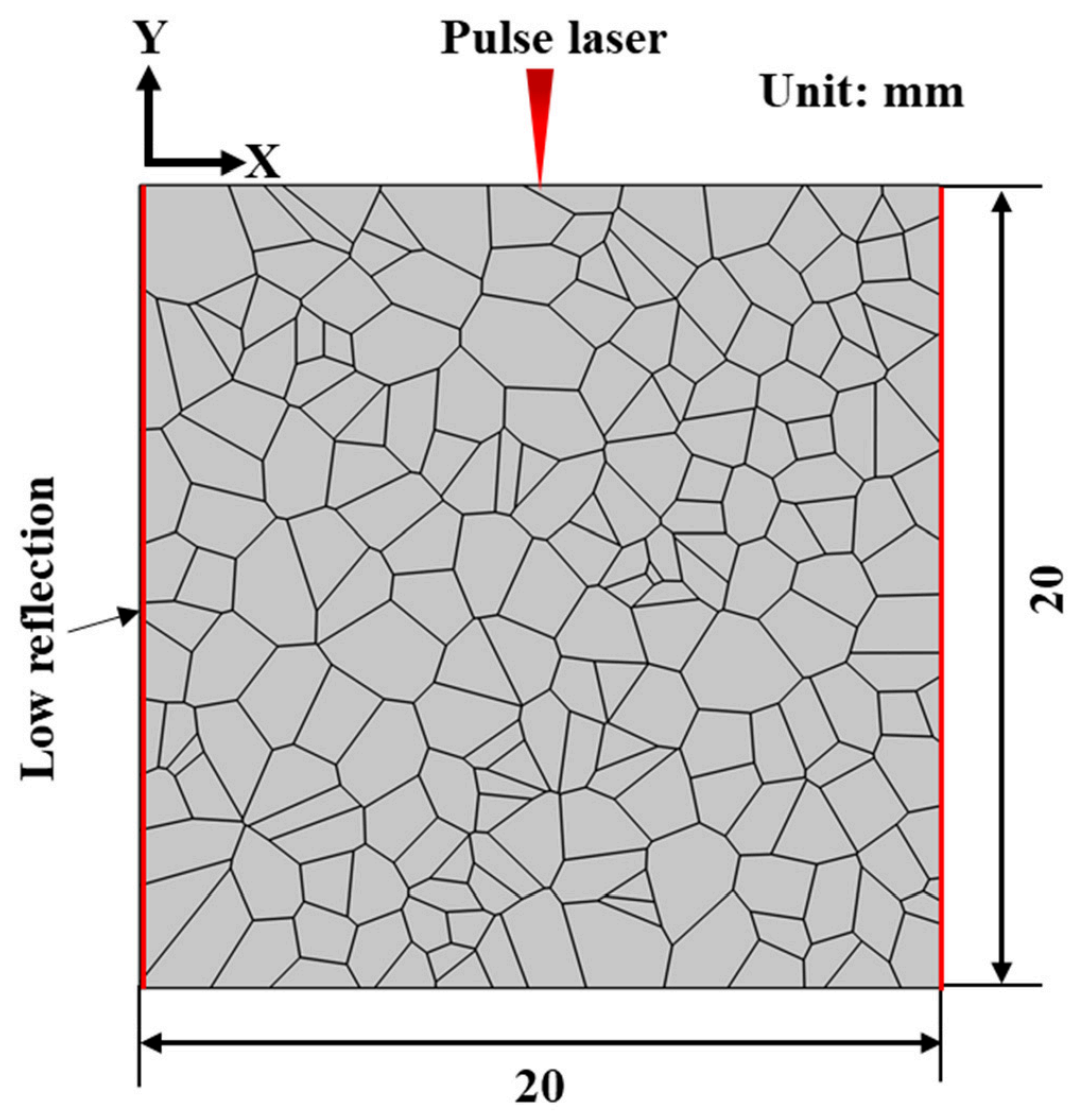
Two-dimensional Voronoi finite element simulation of a polycrystalline model with 200 grains, with low-reflecting boundary conditions (red) applied on the left and right boundaries.

Here, X is a point in X, and $d\left(x,P_{k,i}\right)$ is the Euclidean distance between *x* and $P_{k,i}$. Using the Voronoi algorithm, a model with 200 grains was generated and imported into the simulation software. The process resulted in a two-dimensional polycrystalline geometric model with dimensions of 20 mm × 20 mm and 200 grains, as shown in Figure 7. To ensure realistic wave propagation and minimize boundary effects, the left and right boundaries of the model were configured as low-reflection boundaries. This configuration helps to prevent interference from echo signals originating from the left and right edges of the model. The governing equations for the simulation are as follows:

(8) $$\sigma \cdot n=-d_{i}\frac{\partial u}{\partial t}$$

where n is the boundary direction vector, σ is the stress tensor, $d_{i}$ is the mechanical damping coefficient, and u is the velocity vector.

The elastic constants of anisotropic media are typically specified in the principal coordinate system of the crystal. However, two-dimensional grain diagrams inherently lack the ability to intuitively convey grain orientation information. In practical modeling, the preferred orientations of different grains introduce a rotational relationship between the local and global coordinate systems. To address this, it is essential to use Euler angles and coordinate axis rotation methods to transform the elastic constant tensor into a unified simulation coordinate system. This transformation ensures the accurate assignment of elastic properties to different grain elements. When simulating two-dimensional ultrasonic wave propagation, the stiffness matrix of a grain in the crystal coordinate system can be represented by three independent elastic constants: $C_{11}$, $C_{12}$, and $C_{44}$. These constants are expressed as follows [35]:

(9) $$C=\left(\begin{array}{cccccc} C_{11} & C_{12} & C_{12} & 0 & 0 & 0\\ C_{12} & C_{11} & C_{12} & 0 & 0 & 0\\ C_{12} & C_{12} & C_{11} & 0 & 0 & 0\\ 0 & 0 & 0 & C_{44} & 0 & 0\\ 0 & 0 & 0 & 0 & C_{44} & 0\\ 0 & 0 & 0 & 0 & 0 & C_{44} \end{array}\right)$$

The orientation of a crystal can be characterized by its rotation angles. The Euler angles method is commonly used to define crystal orientation. This method involves three independent rotation angles, $\varphi_{1}$, Φ, and $\varphi_{2}$. When the crystal is rotated using these three Euler angles, the crystal orientation matrix Q can be expressed as follows:

(10) $$\begin{array}{l} Q=\left(\begin{array}{ccc} \cos(\varphi_{1}) & -\sin(\varphi_{1}) & 0\\ \sin(\varphi_{1}) & \cos(\varphi_{1}) & 0\\ 0 & 0 & 1 \end{array}\right)\left(\begin{array}{ccc} \cos(\Phi ) & 0 & \sin(\Phi )\\ 0 & 1 & 0\\ -\sin(\Phi ) & 0 & \cos(\Phi ) \end{array}\right)\left(\begin{array}{ccc} 1 & 0 & 0\\ 0 & \cos(\varphi_{2}) & -\sin(\varphi_{2})\\ 0 & \sin(\varphi_{2}) & \cos(\varphi_{2}) \end{array}\right)\\ \;\;\;=\left(\begin{array}{ccc} \cos(\Phi )\cos(\varphi_{1}) & \;\sin(\varphi_{2})\sin(\Phi )\cos(\varphi_{1})-\cos(\varphi_{2})\sin(\varphi_{1}) & \;\sin(\Phi )\cos(\varphi_{2})\cos(\varphi_{1})+\sin(\varphi_{2})\sin(\varphi_{1})\\ \cos(\Phi )\sin(\varphi_{1}) & \;\sin(\varphi_{2})\sin(\Phi )\sin(\varphi_{1})+\cos(\varphi_{2})\cos(\varphi_{1}) & \;\sin(\Phi )\sin(\varphi_{1})\cos(\varphi_{2})-\sin(\varphi_{2})\cos(\varphi_{1})\\ -\sin(\Phi ) & \;\sin(\varphi_{2})\cos(\Phi ) & \;\cos(\varphi_{2})\cos(\Phi ) \end{array}\right)\\ \;\;\;=\left(\begin{array}{ccc} a_{11} & a_{12} & a_{13}\\ a_{21} & a_{22} & a_{23}\\ a_{31} & a_{32} & a_{33} \end{array}\right) \end{array}$$

In the practical modeling, regional variations in grain-preferred orientations are often observed. This variation results in a mismatch between the local crystal coordinate system and the global simulation coordinate system. Essentially, this mismatch can be viewed as a rotation of the crystal coordinate system about its origin. Therefore, Euler angles and coordinate axis rotation methods are essential for transforming the elastic constant tensor from the crystal’s principal coordinate system to a unified simulation coordinate system. The transformation between these different coordinate systems can be achieved using a rotation matrix, whose expression can be derived from the following matrix [36]:

(11) $$A=\left(\begin{array}{cccccc} a_{11}^{2} & a_{12}^{2} & a_{13}^{2} & 2a_{12}a_{13} & 2a_{11}a_{13} & 2a_{11}a_{12}\\ a_{21}^{2} & a_{22}^{2} & a_{23}^{2} & 2a_{22}a_{23} & 2a_{21}a_{23} & 2a_{21}a_{22}\\ a_{31}^{2} & a_{32}^{2} & a_{33}^{2} & 2a_{32}a_{33} & 2a_{31}a_{33} & 2a_{31}a_{32}\\ a_{21}a_{31} & a_{22}a_{32} & a_{23}a_{33} & a_{22}a_{33}+a_{32}a_{23} & a_{23}a_{31}+a_{33}a_{21} & a_{12}a_{32}+a_{31}a_{22}\\ a_{31}a_{11} & a_{32}a_{12} & a_{33}a_{13} & a_{32}a_{13}+a_{12}a_{33} & a_{33}a_{11}+a_{13}a_{31} & a_{31}a_{12}+a_{11}a_{32}\\ a_{11}a_{21} & a_{12}a_{22} & a_{13}a_{23} & a_{12}a_{23}+a_{22}a_{13} & a_{13}a_{21}+a_{23}a_{11} & a_{11}a_{22}+a_{21}a_{12} \end{array}\right)$$

By applying a matrix transformation, the elastic stiffness matrix C defined in the crystal coordinate system can be rotated into the actual coordinate system. This results in a new elastic stiffness matrix, denoted as $C^{\prime }$:

(12) $$C^{\prime }=ACA^{T}$$

**Figure 8:**
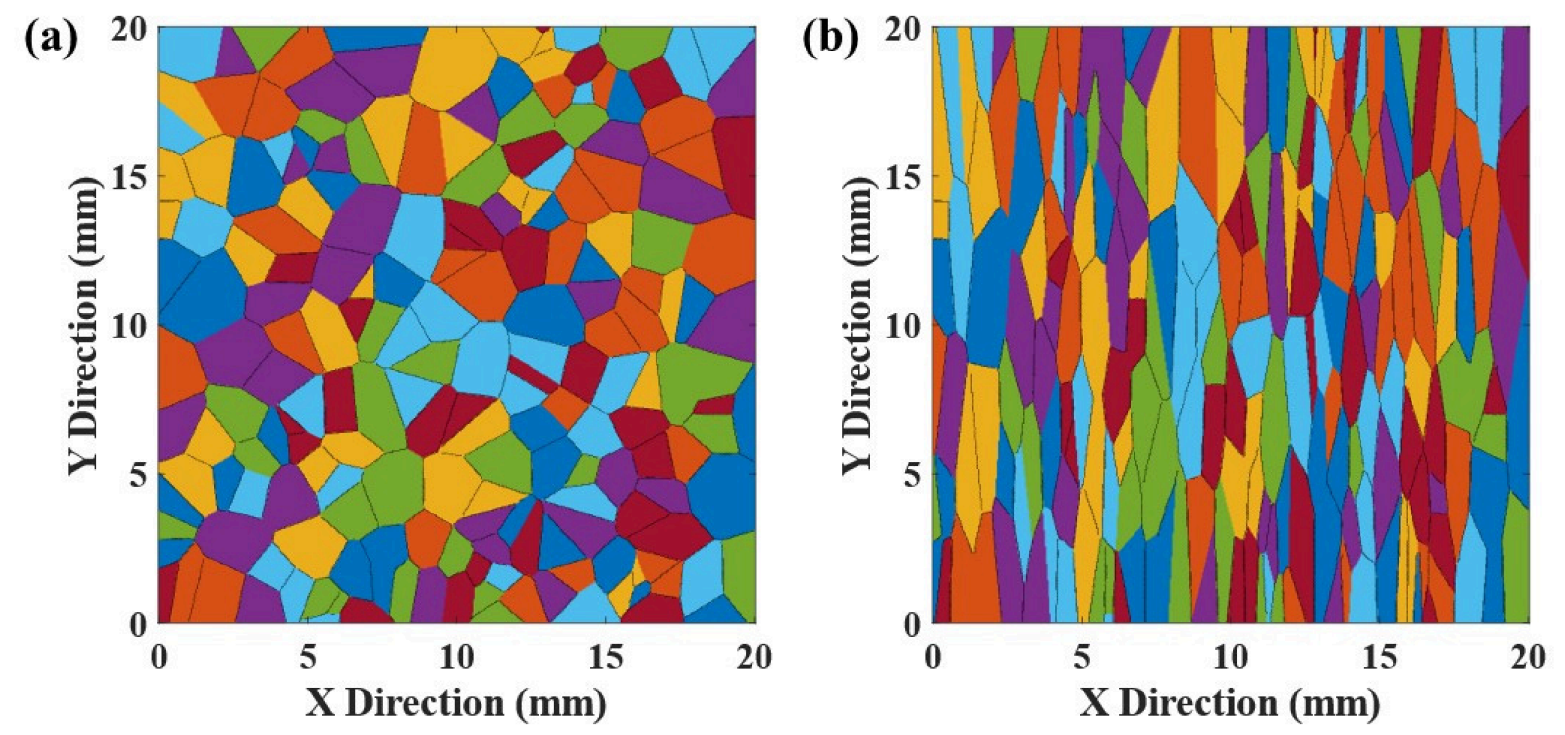
Two-dimensional polycrystalline models established by the Voronoi algorithm (different colors represent different crystal orientations): (**a**) Equiaxial grains, (**b**) Columnar grains.

Then, both isotropic and anisotropic FEM were constructed to characterize additively manufactured Ti6Al4V mechanics. The isotropic model relied on Young’s modulus and Poisson’s ratio. The anisotropic model integrated a grain-scale elastic matrix framework where individual grains received distinct crystallographic orientations and elastic properties. Microstructural observations of LPBF-fabricated Ti6Al4V revealed directional texture. Equiaxed grains formed perpendicular to the deposition direction while coarse columnar grains dominated along the build axis. To replicate this anisotropy, two representative volume element models were generated via Voronoi algorithm. One model represented equiaxed grain structures. The other simulated columnar grains with a 1:5 aspect ratio achieved through coordinate scaling. Integration of these grain-scale models into the elastic material module yielded two-dimensional polycrystalline microstructures, as illustrated in Figure 8.

**Figure 9:**
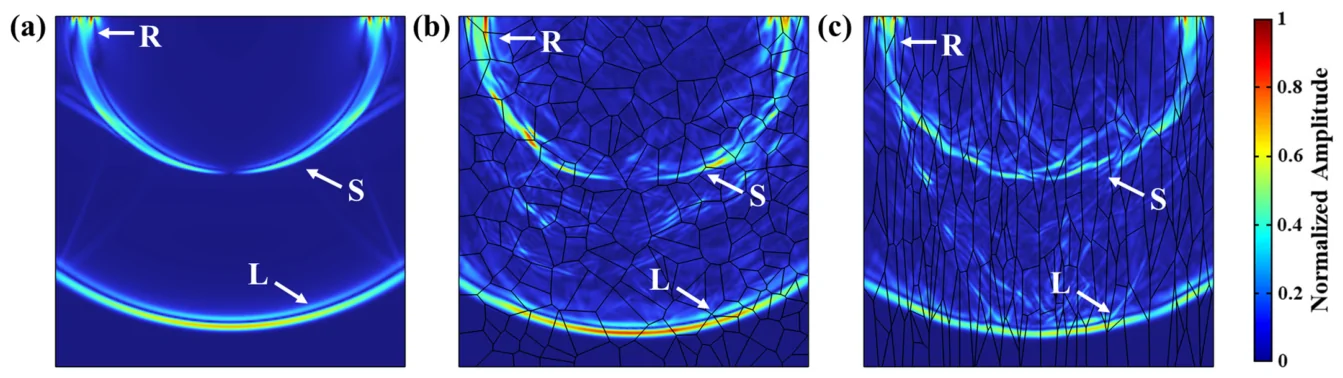
Ultrasonic displacement distribution images under 5 MHz excitation at *t* = 3.0 μs: (**a**) isotropic material, (**b**) equiaxial grains, (**c**) columnar grains.

To investigate microstructural effects on wave propagation, ultrasonic simulations were performed at *t* = 3 μs for each of isotropic, anisotropic equiaxed and anisotropic columnar microstructures. Corresponding displacement fields are presented and compared in Figure 9. Here, R, S and L denote surface, shear and longitudinal waves, respectively. Waveform analysis revealed significant scattering in anisotropic materials. This scattering compromised waveform integrity and produced irregular wave fronts. In contrast, isotropic materials exhibited clear, regular waveforms with minimal scattering. These results underscore distinct propagation characteristics between isotropic and anisotropic materials. The pronounced scattering in anisotropic materials stems from heterogeneous grain structures and varying elastic properties. This finding highlights key challenges for ultrasonic testing of additively manufactured components.

**Figure 10:**
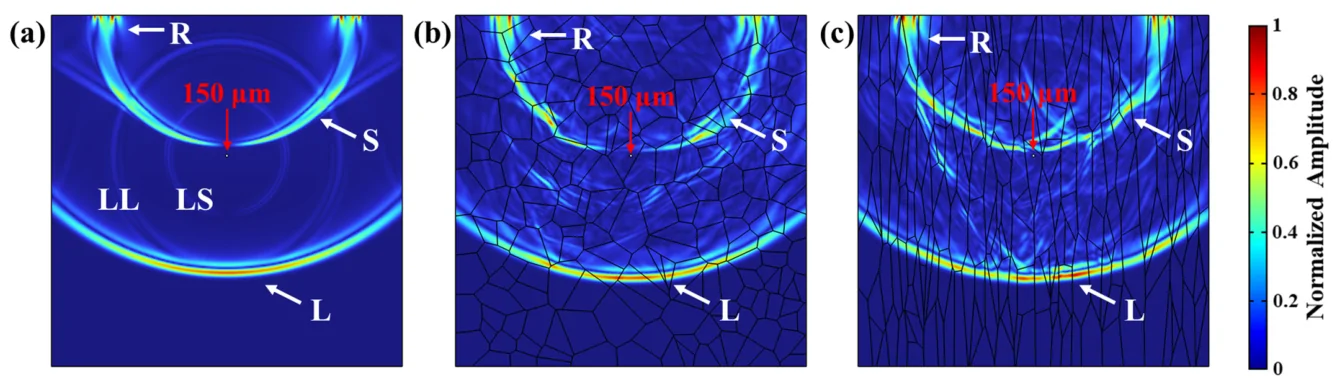
Ultrasonic displacement distribution images at *t* = 2.5 μs with a 150 μm defect: (**a**) isotropic material, (**b**) equiaxial grains, (**c**) columnar grains.

Building on the defect-free baseline simulations, a 150 diameter defect was introduced at the center of the three aforementioned models, positioned 8 mm below the surface. Simulated displacement distributions for the defect-containing configurations at *t* = 2.5 μs are plotted in Figure 10. In the isotropic material, scattered longitudinal (LL) and shear (LS) waves propagate uniformly outward after ultrasonic interaction with the defect, with distinct wave modes clearly resolvable. In contrast, defect-induced scattering is obscured in the two anisotropic material sets by pervasive grain boundary interference.

**Figure 11:**
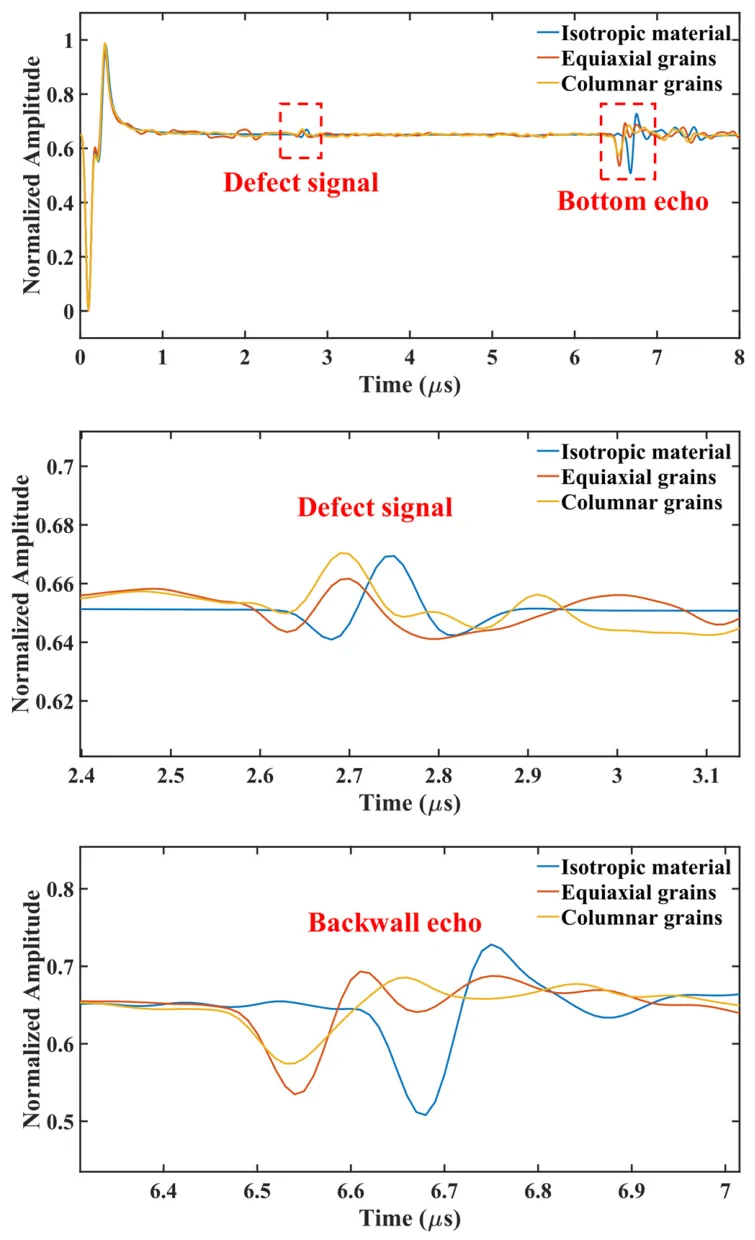
Simulated A-scan signals for isotropic and anisotropic materials with a 150 μm defect, along with magnified views of the defect signal and the backwall wave.

Additionally, these wave propagation differences can be distinguished from displacement A-scan results at *t* = 2.5 μs plotted in Figure 11. In the isotropic material, defect scattering signals appear between the generation wave and backwall wave, with a smooth curve free of interference. In contrast, the two anisotropic material sets exhibit irregular clutter interference (grain noise) alongside defect scattering signals between the generation and backwall waves. Grain noise could mask defect signals, impairing internal microdefect detection. Grain boundary scattering also reduces the amplitude of both defect signals and backwall waves, indicating enhanced scattering attenuation in anisotropic materials and increased defect identification difficulty. When signal amplitude is defined as the difference between the maximum and minimum values of defect signals, Table 2 shows that defect signal amplitudes in the two anisotropic material groups decrease by 36.4% and 29.2%, respectively, compared to the isotropic material. As observed in the defect signal and backwall wave amplification diagrams in Figure 11, signal arrival time in anisotropic materials is slightly shorter than in isotropic materials, suggesting higher ultrasonic wave velocity in anisotropic microstructures.

## 3. Laser Ultrasonic Testing Experiment

### 3.1. Experimental Setup and LPBF Components

**Figure 12:**
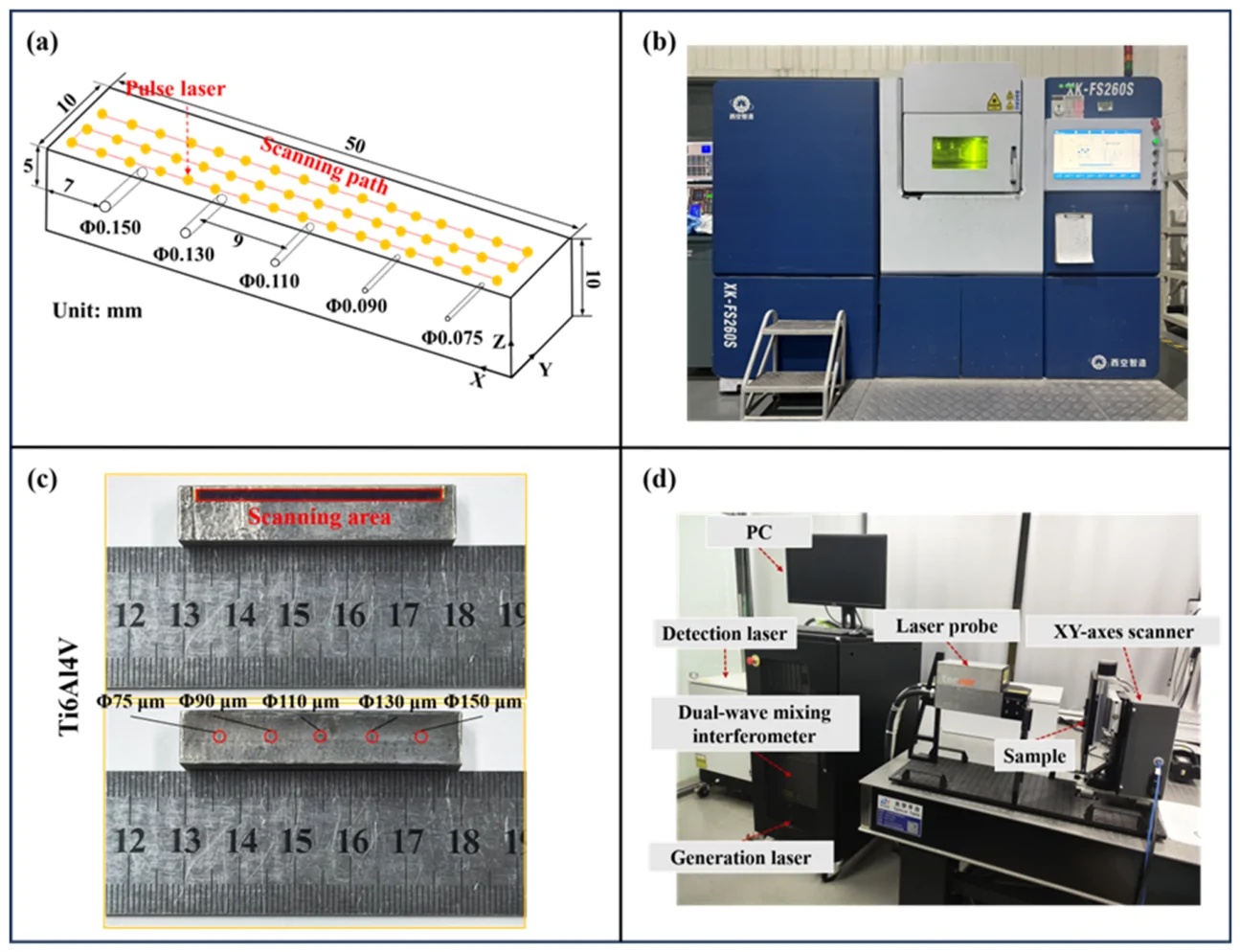
(**a**) Schematic diagram of sample processing, (**b**) LPBF additive manufacturing equipment, (**c**) Ti6Al4V samples and samples with porosity defects produced by LPBF, (**d**) laser ultrasonic testing system.

The overall experimental workflow is illustrated in Figure 12. Figure 12a shows the geometric schematic of the sample with laser ultrasonic scanning configuration, and Figure 12b depicts the LPBF additive manufacturing equipment utilized in this investigation. Figure 12c presents the LPBF-manufactured Ti6Al4V sample together with its laser ultrasonic scanning region, while Figure 12d illustrates the laser ultrasonic testing system. The samples were produced using the XK-FS260S system, with the Ti6Al4V processing parameters listed in Table 3. Rectangular samples of 50 mm × 10 mm × 10 mm were vertically mounted on the scanner platform, and scanning was performed by moving the sample to enable both line and area scans. To simulate micro-damage within the material, artificial hole-type defects with diameters of 75–150 μm were embedded at a depth of 5 mm at equal intervals.

These samples were inspected using the laser ultrasonic detection system described below (Figure 12d). The system comprises an industrial PC, a dual-wave mixing interferometer, a generation laser, a laser probe, a detection laser, and a XY-axes scanner. The generation laser operates at a wavelength of 532 nm and excites ultrasonic waves on the sample surface, whereas the detection laser operates at 1064 nm and is coupled to the dual-wave mixing interferometer, which receives the ultrasonic signals through optical interference. This interferometer is based on a GaAs photorefractive crystal, which employs a dynamic holographic grating to mix signal and reference light, achieving a sensitivity of 2 × 10^−7^ nm (W/Hz)^1/2^. The optical paths of both the generation and detection lasers are integrated within the laser probe, and the generation source and the detection beam are focused on the same side of the sample with spatially coincident generation and detection points.

Consequently, the system operates in reflection mode and scans the sample point-by-point at a detection sampling rate of 125 MHz/s, maintaining a fixed time delay of 1.2 μs between consecutive laser pulses. To ensure coverage of all defect areas, which are marked in Figure 12c, the scan was performed with a step size of 0.10 mm over a 42 mm × 2 mm range.

To ensure reliable and repeatable inspection, the measurements were conducted in the thermoelastic regime, in which a pulsed laser excites ultrasonic waves without damaging the surface. As documented in Table 4, the laser energy density was intentionally calibrated below the material’s surface damage threshold to ensure ultrasonic wave generation via thermal expansion rather than surface ablation. Prior to formal testing, all sample surfaces were systematically ground and polished to achieve a uniform, smooth finish. Multiple preliminary laser ultrasonic scans were then performed to remove residual surface contaminants and validate the repeatability and stability. These pre-experimental steps were implemented to eliminate surface roughness as a confounding variable, ensuring that measured signal variations could be attributed primarily to internal defects rather than topographical irregularities.

**Figure 13:**
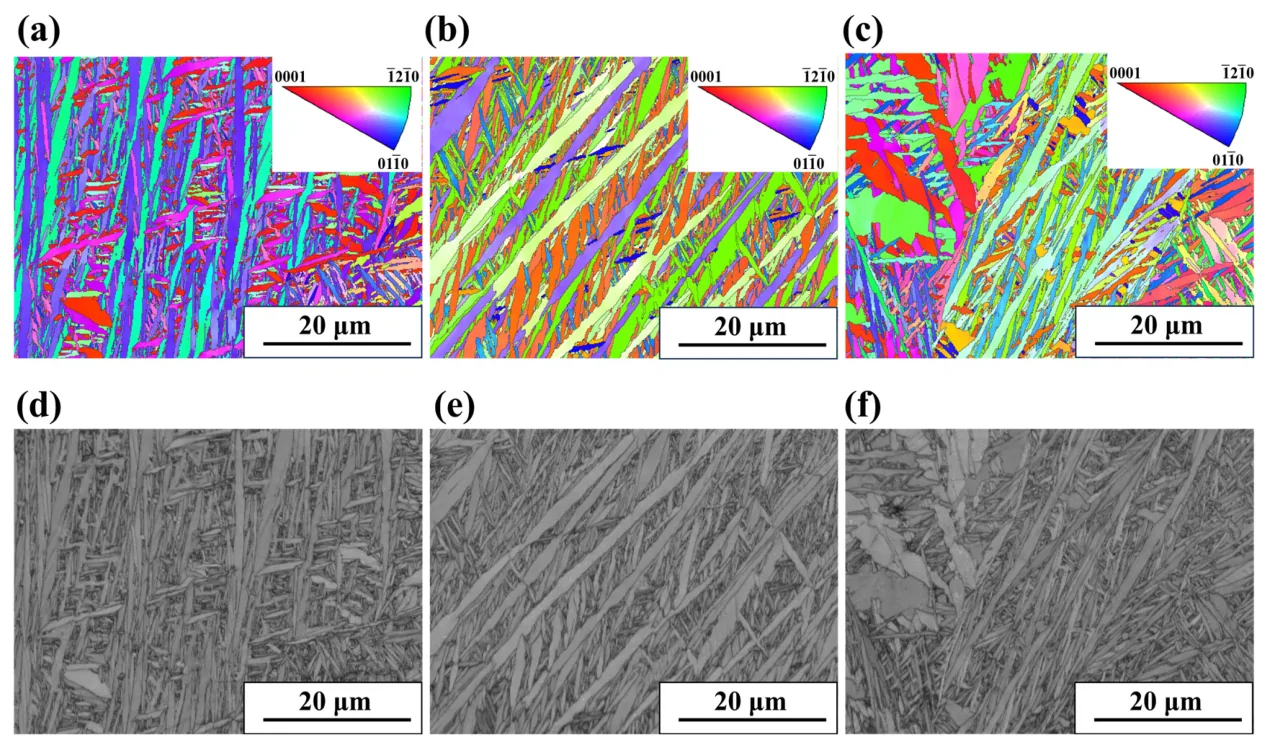
EBSD characterization results of an LPBF Ti6Al4V sample in three orthogonal directions (XY, XZ, YZ): (**a**–**c**) inverse pole figures (IPF) showing grain orientation distribution (different colors represent different crystal orientations) and (**d**–**f**) band contrast (BC) images showing grain morphology and grain boundary distribution.

In order to characterize the actual microstructure of LPBF-fabricated Ti6Al4V samples and validate the grain structure settings in the simulation model, electron backscatter diffraction (EBSD) analysis was performed along three orthogonal directions (XY, XZ, YZ). Figure 13 presents comprehensive EBSD characterization results, where inverse pole figures (IPF) (Figure 13a–c) visualize crystallographic orientation distribution (different colors represent distinct crystal orientations), and band contrast (BC) images (Figure 13d–f) reveal grain morphology and boundary distribution. BC images clearly show that grains in all three directions exhibit acicular and lamellar characteristics which closely match the columnar polycrystalline model employed in the simulation. This confirms that the simulation accurately captures the typical grain structure of LPBF Ti6Al4V. Quantitative analysis of EBSD data yields average grain sizes of 4.85 μm (XY), 4.82 μm (XZ), and 4.98 μm (YZ), with minimal variation across directions. This narrow size distribution validates the simulation’s grain size parameters and ensures ultrasonic scattering behavior is modeled with realistic microstructural dimensions. The XY direction shows a dominant (0110) crystal plane orientation, while the XZ direction exhibits a preferred (1210) crystal plane orientation. In contrast, the YZ direction displays a random orientation distribution. The grain orientations in the three directions exhibit distinct characteristics, reflecting the strong anisotropy of metals produced by additive manufacturing.

### 3.2. Components Principal Component Analysis for Signal Processing

**Figure 14:**
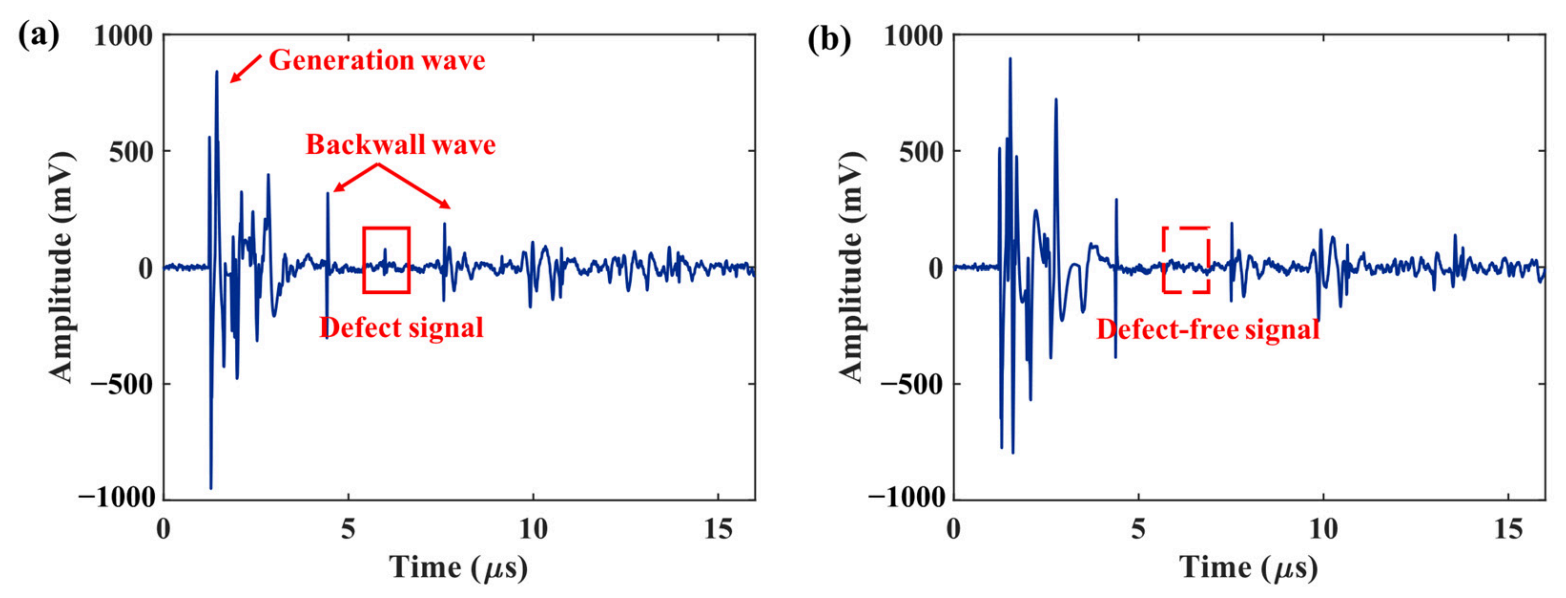
Laser ultrasonic A-scan images: (**a**) defect area and (**b**) defect-free area.

The A-scan image obtained from the laser ultrasonic experiment is shown in Figure 14, which clearly illustrates the differences in ultrasonic signal responses between defect and defect-free regions across the two backwall waves. This single-point time-domain measurement represents superposition of all arriving wave signals, where temporal overlap and material grain noise obscure individual wave peaks despite their physical presence in the sample. As a result, there is a blind zone between the generation wave and the first backwall wave. In laser ultrasonic testing, this blind zone is defined as the time window spanning from laser excitation pulse triggering to the arrival of the first backwall wave, during which defect signals cannot be effectively identified or resolved. Three fundamental causes underline this phenomenon. First, the laser ultrasonic generation wave exhibits high peak intensity and a wide pulse width, typically lasting several microseconds. Its prolonged oscillation decay masks early-arriving scattered or reflected signals from near-surface defects within 1–2 mm of the sample surface. Second, severe multi-mode wave overlap occurs within this time window, with longitudinal, transverse, and surface waves, as well as boundary echoes and multiple mode-conversion waves, arriving simultaneously to form a complex superimposed waveform that drowns out weak microdefect echoes in the early time domain. Third, grain-boundary scattering introduces additional noise that further hinders detection of faint microdefect signals. To mitigate such noise, principal component analysis (PCA)-based signal decomposition methods are considered.

Among these methods, the principal component subtraction (PCS) approach proposed in Reference [29] performs eigenvalue decomposition on the full-time-domain signal matrix, attributing strong-amplitude interference such as surface-skimming longitudinal waves, surface waves, and their boundary echoes to the largest eigenvalues. Removing the first *k* largest eigenvalues suppresses these coherent noises, enabling near-surface defect detection when combined with the synthetic aperture focusing technique (SAFT). This “strong-interference-dominant” method is predicated on the assumption that interference eigenvalues are substantially greater in magnitude than those associated with defects and noise.

However, detecting internal microdefect detection in additively manufactured alloys faces a different acoustic environment. Coarse grains scattering noise is the dominant interference, with amplitude comparable to weak defect signals but weaker spatial correlation. Additionally, the blind zone between the generation wave and the first backwall wave prevents simple time-domain defect selection. Directly discarding the largest eigenvalues, as in PCS, would likely eliminate weak defect signals as well.

We therefore propose a time-truncation principal component retention denoising strategy: only the backscattered signals between the first and second backwall waves are extracted for PCA. In this window, the blind-zone interference is avoided, and the defect-scattered signals (stronger spatial correlation than the random grain noise) correspond to larger eigenvalues. Hence, the first *r* largest eigenvalues are retained to the signal reconstruction, while the small eigenvalues representing random noise are discarded. This operation is opposite to the “large-eigenvalue subtraction” in PCS, shifting from eliminating strong coherent interference to extracting weak coherent defect components. Finally, the denoised signals are used for SAFT imaging of internal defects in LPBF-manufactured Ti6Al4V by laser ultrasonics.

**Figure 15:**
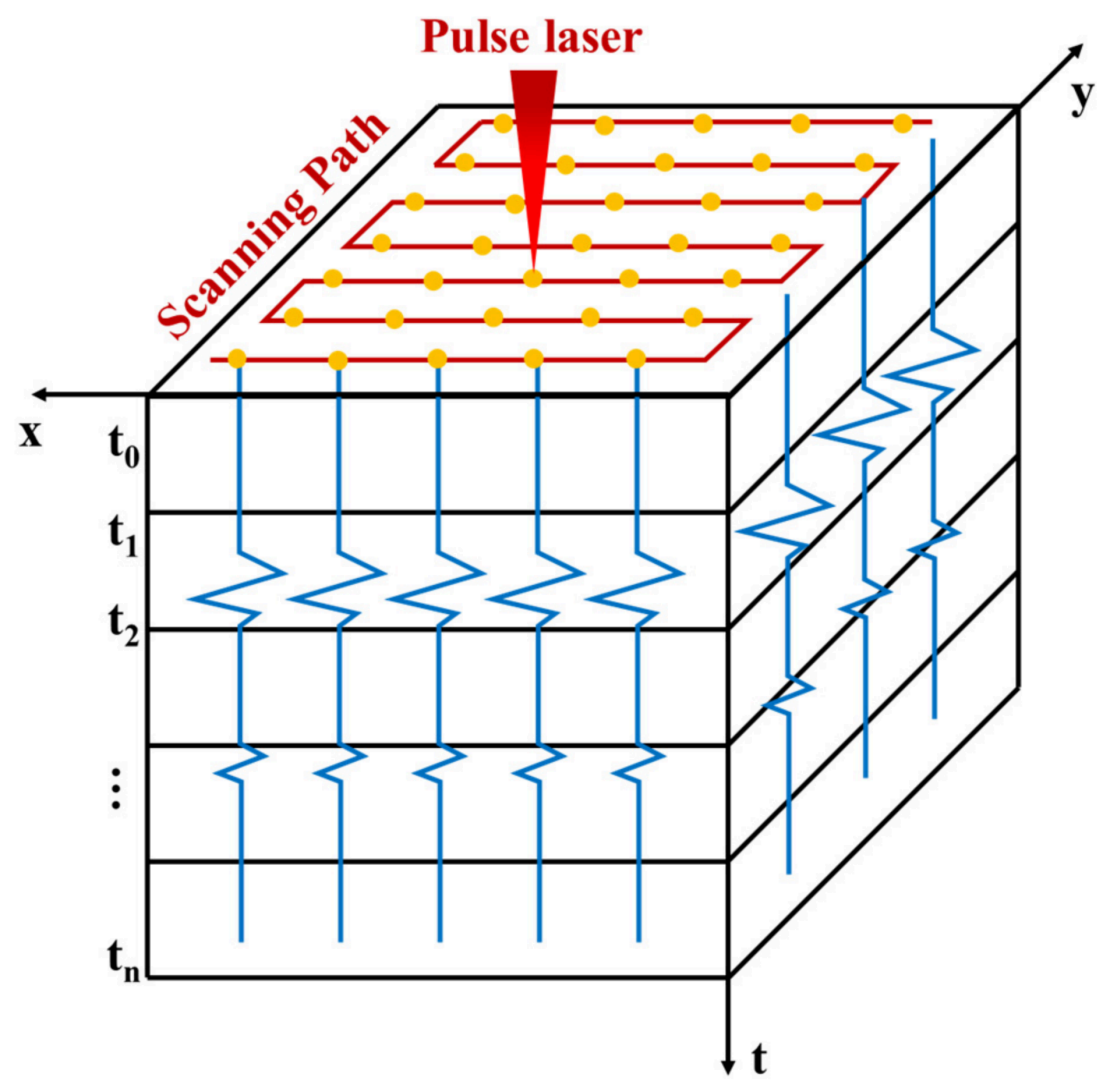
Storage of 3D laser-ultrasound data.

Principal component analysis (PCA), a classic method for data dimensionality reduction, was employed to process the signals and reduce noise interference. PCA decomposes a signal into several orthogonal principal components, suppressing noise by eliminating the dominant background components. Laser ultrasound performs a two-dimensional, point-by-point scan across the surface of the specimen (i.e., the x-y plane), as shown in Figure 15. For all points on the scanned surface, the interferometer acquires the laser ultrasound time-domain signals and stores them in a three-dimensional matrix U(M,N,T), where M and N represent the number of scanning points in the transverse (*x* direction) and longitudinal (*y* direction) directions, respectively, and T represents the signal sampling duration (from $t_{0}$ to $t_{n}$).

To perform PCA, first transform the three-dimensional matrix signal U into a two-dimensional matrix signal $U\left(S,T\right)$, where S=M×N. PCA then performs an eigenvalue decomposition on the covariance matrix to obtain a set of orthogonal basis vectors (principal components). The projection of the data onto the first r principal components can be used to reconstruct the signal and filter out noise. First, we perform a decentering operation on the two-dimensional matrix U:

(13) $$U^{\prime }=U-\mu$$

where $U^{\prime }$ denotes the decentralized matrix, and μ represents the average of the columns containing the matrix elements. Subsequently, the eigenvectors and eigenvalues of the signal matrix are obtained via eigenvalue decomposition:

(14) $$Cov\left(U^{\prime }\right)E=ED$$

in which E is a matrix composed of the eigenvectors of the covariance matrix, and D is a diagonal matrix whose diagonal elements correspond to the eigenvalues. In ultrasonic signals, noise signals typically contribute little to the principal components. Therefore, smaller eigenvalues can be discarded to achieve noise reduction:

(15) $$E_{c}=E_{s}\left(:,1:r\right)$$

Here, $E_{c}$ denotes the matrix of filtered feature vectors, $E_{s}$ denotes the matrix of feature vectors sorted in descending order, and r denotes the retention threshold, indicating that the top r most discriminative features are extracted from all A-scan signals.

Global PCA applied to ultrasonic signals can include excessive data from non-defective intervals, leading the principal components to deviate from defect features. To address this, a time-truncation PCA strategy is employed, focusing on the defect echo range between the first and second backwall waves. This strategy minimizes the influence of non-defective intervals and enhances the variance contribution of defect signals in the local region, enabling more precise extraction of defect information. The detection blind zone exists between the generation wave and the first echo and complicates the defect echoes’ identification. This ensures that the principal components better reflect the characteristics of defects, thus enhancing the overall quality of the analysis.

### 3.3. Experimental Results and Analysis

Referring back to Figure 14, A-scan images from defect and non-defect regions are compared, clearly showing a defect echo signal around 6.0 μs. The primary analysis interval is the backscatter signal region between the first and second backwall waves (4.7–7.2 μs).

For experimental SAFT reconstruction, longitudinal wave velocity was measured directly from laser ultrasonic A-scan signals using a five-point calibration method. Five measurement points were selected in defect-free areas to record sample thickness *d* and the first backwall wave arrival time *t*_1_. Velocity was calculated for each point using $c_{L}$ = 2*d*/*t*_1_, yielding an average value of 6365 m/s, which exceeds the simulated anisotropic material value of 6211 m/s by approximately 2.4%. This discrepancy is attributed to strong crystallographic anisotropy inherent in additively manufactured Ti6Al4V, as confirmed by EBSD texture analysis results presented in Figure 13. This calibrated velocity of 6365 m/s was used for all experimental SAFT reconstructions to ensure accurate defect localization.

**Figure 16:**
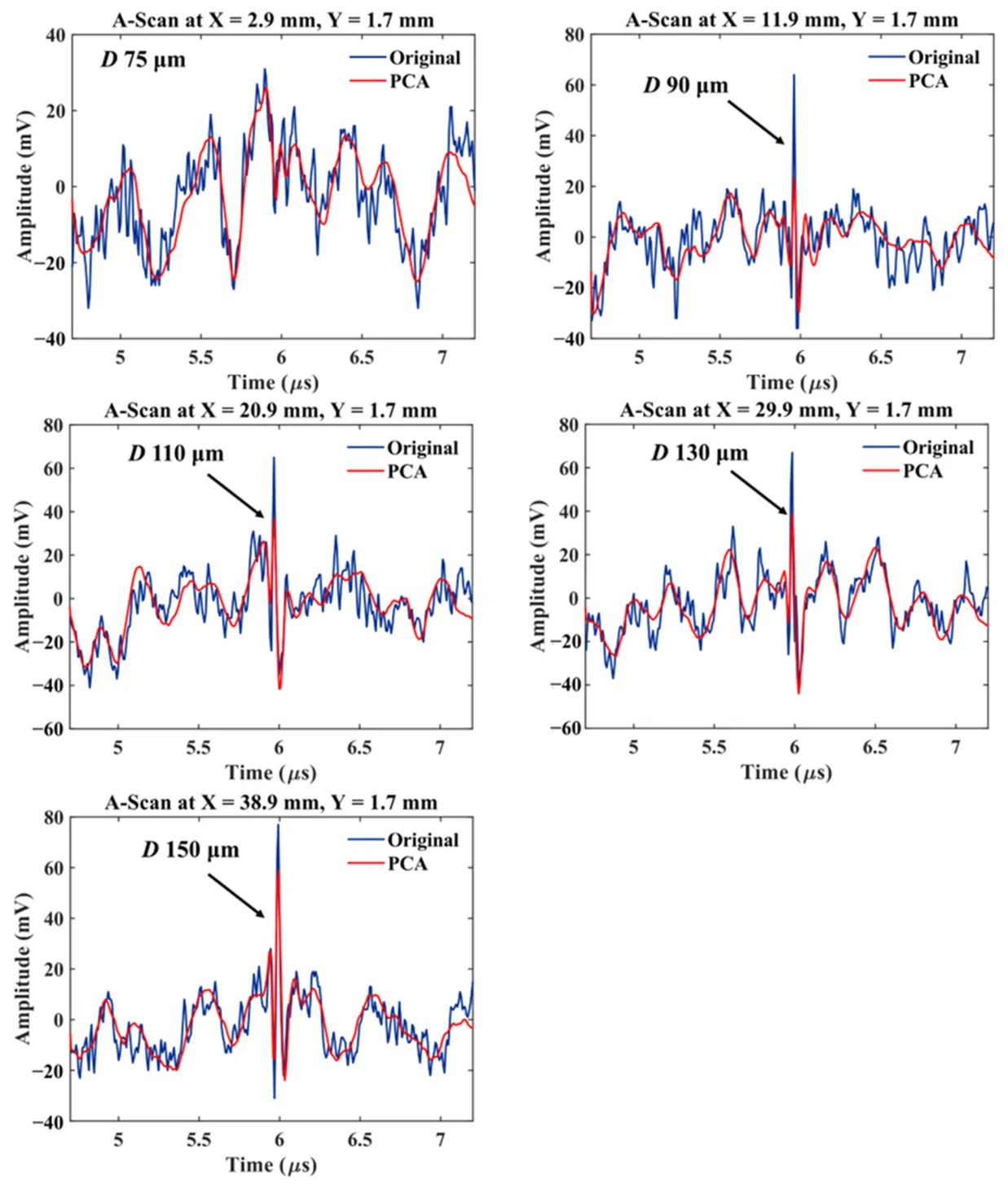
A-scan signals at defect locations before and after PCA-based noise reduction.

Figure 16 displays A-scan signals within this time range, along with their PCA-denoised results, for five defect sizes from 75 to 150 μm. The original signals contain notable high-frequency noise, obscuring the 75 μm defect echo. After PCA denoising, the signal quality improves measurably, preserving local features and clarifying waveform contours. However, the 75 μm defect signal remains undetectable, indicating limitations in microdefect detection under current conditions.

**Figure 17:**
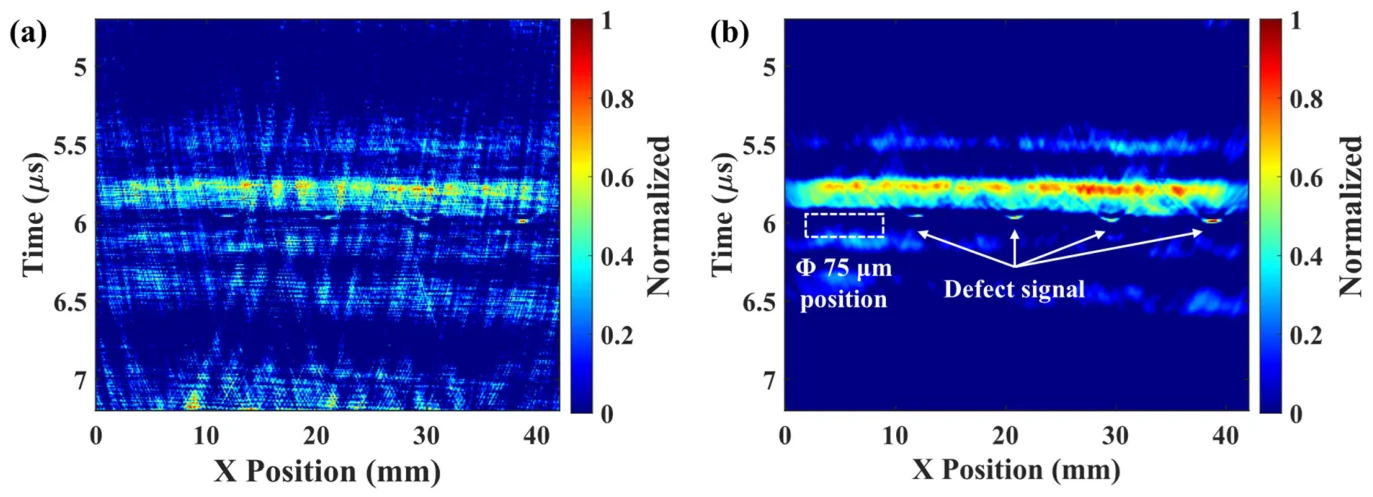
Laser ultrasonic SAFT imaging: (**a**) original signal and (**b**) signal after PCA processing.

In laser ultrasound experiments, column signals from single scans were processed using SAFT, with the results shown in Figure 17. Figure 17a shows SAFT reconstruction on original data, exhibiting discrete speckle noise, blurred defect contours, and low contrast, hindering accurate defect identification. We apply PCA for noise reduction prior to SAFT imaging, with the results shown in Figure 17b. Compared to the original SAFT image (Figure 17b), this improves the imaging quality. The PCA-SAFT image shows defects as focused bright spots, with suppressed background noise and imaging resolution. At the white arrows in Figure 17b, continuous and well-focused signals corresponding to 90 to 150 μm defects are observed, demonstrating PCA’s effectiveness in reducing noise and enhancing the SAFT focusing.

**Figure 18:**
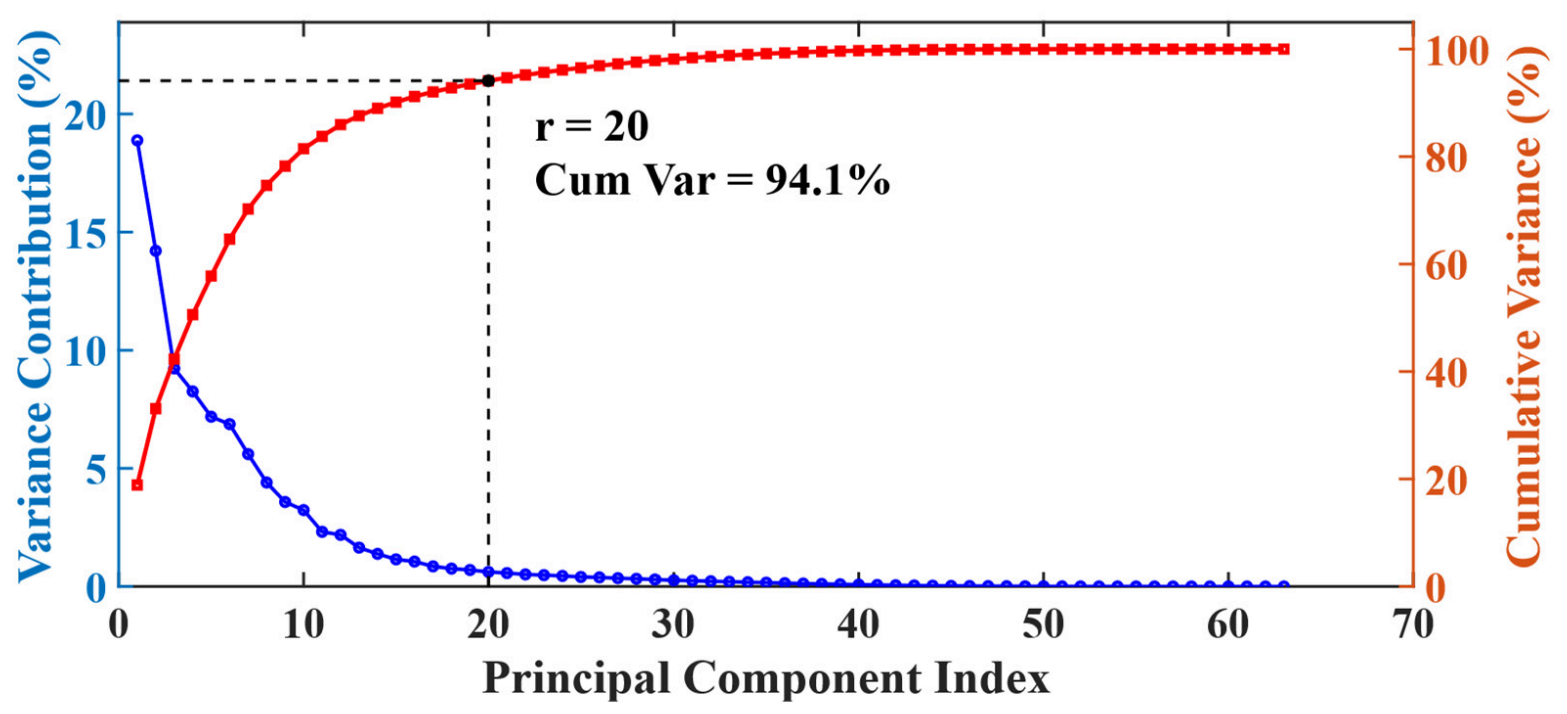
Variance contribution of principal component eigenvalues.

Combining the PCA-SAFT results, the defect signal is primarily concentrated within the 5.7–6.2 μs time range. A variance-based criterion is established to determine the number of retained principal components (*r*). This criterion leverages the principle that defect signals exhibit stronger spatial correlation than random grain noise. The stronger correlation leads to variance concentration in the first few principal components while noise components distribute across higher-order components. A cumulative variance contribution threshold of 85–95% is set to separate signal from noise. For the time-truncation range of 5.7–6.2 μs, this threshold corresponds to *r * = 20. Principal components beyond this threshold primarily represent random grain scattering and system noise. These components are discarded to avoid introducing irrelevant information. Figure 18 visually demonstrates eigenvalue information distribution during PCA dimensionality reduction. The horizontal axis shows principal component indices sorted by descending eigenvalues. Lower indices indicate higher information content. The blue line with circular markers represents individual component variance contribution rates. The red line with square markers shows cumulative variance contribution rates. This figure provides an intuitive reference for the threshold selection rationale.

**Figure 19:**
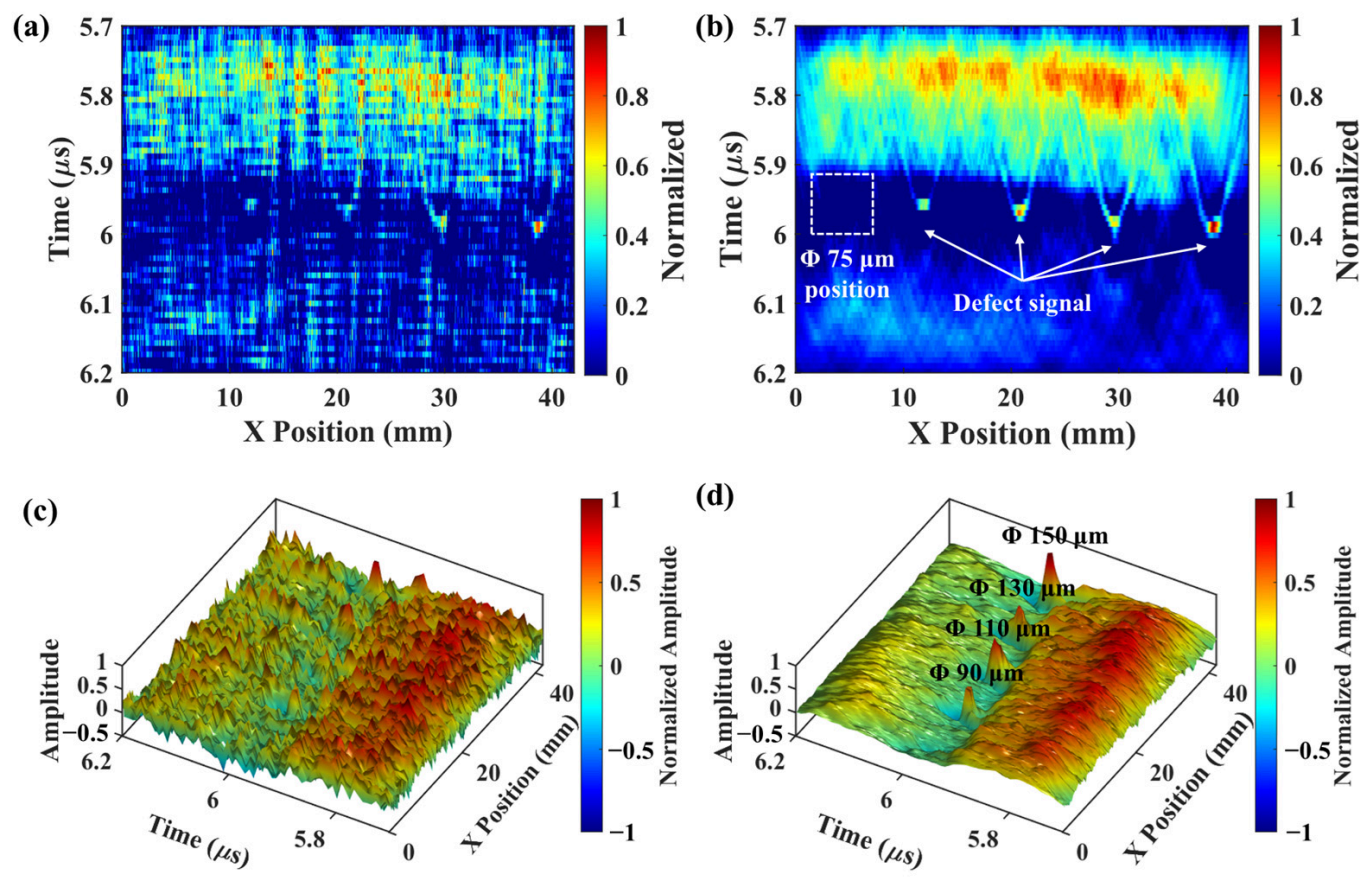
SAFT imaging results before and after fine PCA processing: (**a**) 2D imaging of the original signal, (**b**) 2D imaging after PCA processing, (**c**) 3D imaging of the original signal, (**d**) 3D imaging after PCA processing.

Narrowing the analysis interval to this range yields the 2D and 3D SAFT imaging results before and after PCA processing, as shown in Figure 19. PCA processing enhances the defect signal and suppresses noise. The 2D image in Figure 19b shows a bright spot with concentrated energy, and the 3D image in Figure 19d shows a spiky protrusion far greater than background noise. These results validate the method’s feasibility for defect visualization. Notably, parabolic-like signals appear near pore defects in Figure 19b, which correspond to diffraction waves generated at defect edges. During SAFT reconstruction, these diffraction waves fail to fully converge at the focal point due to their complex propagation paths, remaining in the image as arc-shaped trails. This artifact is particularly pronounced when the signal-to-noise ratio is insufficient. While PCA effectively suppresses random noise, it cannot completely separate diffraction waves that share similar statistical characteristics with true defect signals. This limitation leads to the retention of these residual artifacts.

To quantify the optimization degree of the laser ultrasonic detection results achieved by the PCA-SAFT method, the contrast-to-noise ratio (*CNR*) is introduced, with its calculation method expressed in Equation (16):

(16) $$CNR=\frac{\left|\mu_{d}-\mu_{bg}\right|}{\sigma_{bg}}$$

where $\mu_{d}$ denotes the average of the absolute pixel amplitudes in the defect region, $\mu_{bg}$ denotes the average absolute pixel amplitudes in the background region (entire SAFT image, excluding the defect mask), and $\sigma_{bg}$ denotes the standard deviation of the absolute pixel amplitudes in the background region.

To ensure statistical robustness, mean CNR values were calculated across five representative defect regions. Prior to PCA processing, the SAFT imaging yielded a mean CNR of 0.94—a value below 1, indicating that noise overwhelmed the defect signal due to inadequate signal-to-noise separation. After applying time-truncated PCA, the mean CNR increased to 1.26, representing a 34% improvement. This quantifiable enhancement provides clear evidence that the PCA-SAFT method effectively boosts defect detection sensitivity and improves the imaging quality.

Laser ultrasonic testing effectively detects 90–150 μm defects in LPBF-manufactured Ti6Al4V alloys but not smaller (75 μm) defects. This may be due to the 0.10 mm laser scanning step size, which limits the imaging system’s ability to capture minute defects, and the resolution of laser-assisted ultrasonic testing. For defects much smaller than the wavelength, most ultrasonic energy passes through, resulting in weak defect scattering. Although PCA-SAFT processing can suppress random noise, coherent enhancement remains challenging for these low-SNR signals, as the method relies on sufficient signal amplitude to achieve constructive interference. These factors collectively impose fundamental limits on detecting sub-wavelength microdefects in additively manufactured materials.

In this study, idealized cylindrical hole defects were selected to isolate the relationship between defect size and ultrasonic signal amplitude, establishing a baseline validation of the PCA-SAFT method’s core capabilities without the confounding variables of complex defect geometries. For crack-like and elongated lack-of-fusion defects—characterized by sharp edges and extended planar dimensions—the method is theoretically well-suited to achieve high detection sensitivity due to its ability to coherently sum directional scattered signals, though performance may degrade if defect orientation is perpendicular to ultrasonic wave propagation. For clustered porosity, while individual sub-resolution pores may not be resolved, the method can detect and localize clusters as aggregated features, with sensitivity being dependent on total cluster cross-sectional area and inter-pore spacing. Critically, the immediate next phase of this research will focus on systematic simulation and experimental studies targeting these real-world defect morphologies, including parametric analyses of the defect orientation, aspect ratio, and spatial distribution, to directly address the limitations of idealized geometries and enhance the method’s characterization capabilities for industrial additively manufactured components.

## 4. Conclusions

This study has thoroughly investigated the detection of internal microdefects in laser powder bed fusion (LPBF)-produced Ti6Al4V alloys using laser ultrasonic finite element analysis and experiments. The research explored defect response characteristics and signal enhancement methods, focusing on the impact of varying microdefect sizes on laser ultrasonic detection. The findings can be summarized as follows:

(1) Enhanced detection with SAFT: In the simulation with defects ranging from 75 to 150 μm, the initial A-scan results showed weak defect echo amplitudes compared to the generation wave and the backwall waves. However, after applying the synthetic aperture focusing technique (SAFT), clear and bright spots emerged at defect locations. This demonstrated the SAFT’s significant advantages in enhancing microdefects’ scattering signals and improving the image signal-to-noise ratio (SNR).
(2) Polycrystalline microstructure impact: A polycrystalline model with varying grain orientations, constructed using the Voronoi algorithm, was used to simulate the complex microstructural characteristics of additively manufactured metals. The study revealed that the polycrystalline microstructure of additively manufactured Ti6Al4V affects ultrasonic wave propagation, with grain scattering identified as the primary source of detection noise. This provides a robust theoretical foundation for future research on microdefect detection and imaging.
(3) Experimental validation and limitations: Laser ultrasonic testing successfully detected the defect—a 90 μm-diameter defect at a depth of 5 mm in the Ti6Al4V alloy. The combination of the proposed time-truncation principal component retention denoising strategy and SAFT effectively reduced noise and improved the imaging quality. However, the inability to detect a 75 μm defect reveals the current detection limits under the experimental conditions.

## Figures and Tables

**Table 1:** Physical properties of Ti6Al4V utilized in the simulation [31,32].

Physical Properties	Density ρ (kg/m^3^)	Young’s Modulus E (GPa)	Poisson’s Ratio ν	Elastic Constant $C_{11}$ (GPa)	Elastic Constant $C_{12}$ (GPa)	Elastic Constant $C_{44}$ (Gpa)
Value	4420	110	0.33	160	90	45

**Table 2:** Amplitudes of defect signals in isotropic and anisotropic materials and their reduction rates.

Material	Defect Signal Amplitude (nm)	Reduction Rate (%)
Isotropic material	10.43	
Equiaxial grains	6.63	36.4
Columnar grains	7.38	29.2

**Table 3:** LPBF additive manufacturing process parameters for Ti6Al4V alloy.

LPBF Process Parameters	Laser Power (W)	Scan Speed (m/s)	Scan Interval (mm)	Bed Layer Thickness (μm)
Value	340	1300	0.11	60

**Table 4:** Parameters of the laser ultrasonic inspection system.

Generation Laser Parameters	Value	Detection Laser Parameters	Value	Other Parameters	Value
Wavelength (nm)	532	Wavelength (nm)	1064	Detection range (mm)	180
Laser beam diameter (mm)	0.5	Laser energy (mJ)	74	Scan step size (mm)	0.10
Laser energy (mJ)	30	Pulse width (μs)	85	Sampling bandwidth (MHz)	60
Pulse width (ns)	8	Peak power (W)	358	Sampling rate (MHz/s)	125

## Data Availability

The original contributions presented in this study are included in the article. Further inquiries can be directed to the corresponding authors.
